# Molecular mechanisms creating bistable switches at cell cycle transitions

**DOI:** 10.1098/rsob.120179

**Published:** 2013-03

**Authors:** Anael Verdugo, P. K. Vinod, John J. Tyson, Bela Novak

**Affiliations:** 1 Department of Biochemistry, University of Oxford, Oxford OX1 3QU, UK; 2 Department of Biological Sciences, Virginia Tech, Blacksburg, VA 24061, USA

**Keywords:** cell cycle, checkpoints, bistability, network motifs

## Abstract

Progression through the eukaryotic cell cycle is characterized by specific transitions, where cells move irreversibly from stage *i*−1 of the cycle into stage *i*. These irreversible cell cycle transitions are regulated by underlying bistable switches, which share some common features. An inhibitory protein stalls progression, and an activatory protein promotes progression. The inhibitor and activator are locked in a double-negative feedback loop, creating a one-way toggle switch that guarantees an irreversible commitment to move forward through the cell cycle, and it opposes regression from stage *i* to stage *i*−1. In many cases, the activator is an enzyme that modifies the inhibitor in multiple steps, whereas the hypo-modified inhibitor binds strongly to the activator and resists its enzymatic activity. These interactions are the basis of a reaction motif that provides a simple and generic account of many characteristic properties of cell cycle transitions. To demonstrate this assertion, we apply the motif in detail to the G1/S transition in budding yeast and to the mitotic checkpoint in mammalian cells. Variations of the motif might support irreversible cellular decision-making in other contexts.

## 2. Introduction

During the cell division cycle, a cell's DNA molecules are accurately replicated and then each pair of identical DNA molecules are carefully partitioned to the pair of newly forming daughter cells, so that each daughter cell gets one and only one copy of each DNA molecule. The perpetuation of life demands that these processes be carried out with nearly flawless precision, although some errors are unavoidable, of course. In addition to the complex biochemical and mechanical mechanisms that carry out DNA replication and cell division, cells use subtle biochemical regulatory networks to ensure the proper completion and sequencing of cell cycle events.

In eukaryotic cells, the cell cycle regulatory network is based on transcriptional and post-translational controls of a class of cyclin-dependent protein kinases (CDKs). As cells proceed periodically through the classic phases of the cell cycle (G1, S, G2, M, G1, …), these CDKs and their associated regulators are periodically activated and inactivated, thereby driving progression from one stage of the cycle to the next. Proper progression through the cell cycle requires that stage *i* must not begin until stage *i*−1 is successfully completed and that the transition from stage *i*−1 to stage *i* is irreversible.

**Figure 1.**
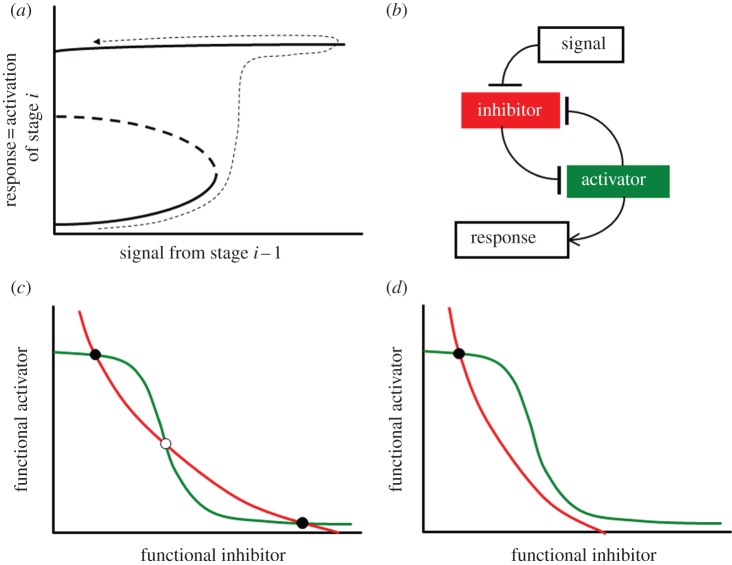
Generic picture of a bistable cell cycle switch. (*a*) Signal–response curve. The activator of stage *i* of the cell cycle (‘response’) is a bistable function of a ‘signal’ generated by stage *i*−1, for 0 ≤ signal < threshold. When the signal exceeds the threshold, the activator turns on and the cell proceeds to stage *i*. Subsequently, even if the signal drops to zero, the activator stays on and the cell remains in stage *i*. (*b*) An activator–inhibitor pair involved in a double-negative feedback loop can generate the sort of bistable response postulated in (*a*). (*c*,*d*) Phase planes for an activator–inhibitor system for (*c*) signal < threshold and for (*d*) signal > threshold.

In general, there are five major transitions at boundaries between sequential stages of the cell cycle in eukaryotes: G1a/G1b (the ‘restriction point’ in mammalian cells, ‘Start’ in yeast), G1/S (initiation of DNA synthesis), G2/M (entry into mitosis), metaphase/anaphase (chromosome alignment on the mitotic spindle) and telophase/G1 (exit from mitosis and return to G1 phase). Progression through each of these transitions is controlled by a balance between inhibitors and activators (table 1) [1]. Before the transition, the inhibitor overwhelms the activator. As soon as the prior stage is judged to be complete, a signal helps the activator to eliminate the inhibitor and the transition occurs. The transitions are irreversible because each biochemical mechanism functions as a ‘bistable’ switch (figure 1*a*). The connection between irreversible cell cycle transitions and bistable biochemical switches has been emphasized repeatedly in the theoretical literature [2–5] and has been confirmed experimentally in all cases [6–11].

**Table 1.** Activators and inhibitors of certain cell cycle transitions. S, stoichiometric inhibitor; E, enzymatic inhibitor.

transition	activator	inhibitor
restriction point	E2F transcription factor	retinoblastoma protein (S)
Start (yeast)	SBF transcription factor	Whi5 (S)
G1/S (yeast)	Clb5-dependent kinase	Sic1 (S)
G1/S (yeast)	Clb5-dependent kinase	Cdh1 (E)
G2/M	cyclin B-dependent kinase	Wee1 (E)
metaphase/anaphase	anaphase-promoting complex	mitotic checkpoint complex (S)

In each case, the bistable switch could potentially provide ‘checkpoint’ control over the cell cycle transition. By monitoring defective states of chromosomes (damaged, not fully replicated, not fully aligned on the mitotic spindle), intracellular surveillance mechanisms could keep the signal (figure 1*a*) at a subthreshold level, thereby blocking the transition. When the defect is repaired, the signal is released to drive the transition. In any given cell type, only a few of the five cell cycle transitions function as ‘checkpoints’ in this sense. But each checkpoint mechanism (we propose) relies on the bistability of the underlying transition.

Bistability at cell cycle transitions is the consequence of a double-negative feedback loop by which the activator inactivates its own inhibitor (figure 1*b*). To illustrate the connection between double-negative feedback loops and bistability/irreversibility, we provide two generic ‘phase–plane diagrams’ in figure 1*c,d* (see the electronic supplementary material, §S4 for a description of how to interpret phase plane diagrams). When signal = 0 (figure 1*c*), there are two stable steady states of the control system: ‘checkpoint engaged’ (lots of inhibitor, very little functional activator), and ‘checkpoint disengaged’ (very little inhibitor, lots of activator). At the beginning of the process, the signal is off and the checkpoint is engaged. As the signal increases from zero towards the threshold, the level of functional activator increases only a little (figure 1*a*), and the checkpoint remains engaged. As soon as the signal exceeds the threshold, the lower steady state (checkpoint engaged) disappears, and the control system is forced to transition to the upper steady state (checkpoint disengaged), as in figure 1*d*. After the transition, even if the signal drops to zero (i.e. return to figure 1*c*), the checkpoint mechanism remains locked in the stable, disengaged steady state. In this sense, the transition is irreversible.

Each of the five cell cycle transitions mentioned previously is controlled by its own unique biochemical mechanism. In some cases, the inhibitor is an enzyme. For example, at the G2/M transition, the protein kinase Wee1 phosphorylates and inhibits the mitotic M-CDK. During the transition, Wee1 gets multiply phosphorylated and inactivated by the active (dephosphorylated) form of the M-CDK [12].

In other cases, a stoichiometric inhibitor binds to the activator. For example, before the restriction point in mammalian cells, the retinoblastoma protein (RB) binds to and inhibits members of the E2F family of transcription factors [13]. As cells pass the restriction point, some E2F molecules escape inhibition by RB and begin to activate expression of the *CYCE* gene. The rise of cyclin E-dependent kinase hyper-phosphorylates/inactivates RB, thereby instantiating the regulatory motif in figure 1*b*. The same is true at Start in yeast. Before Start, Whi5 binds to and inhibits the transcription factor, SBF. At the transition point, SBF turns on production of the G1 cyclins, Cln1 and Cln2, and the Cln-dependent kinases phosphorylate and neutralize Whi5 [14,15].

The G1/S transition is also controlled by a stoichiometric inhibitor: p27^Kip1^ in mammalian cells, p40^Sic1^ in budding yeast and p25^Rum1^ in fission yeast [16]; generically known as cyclin-dependent kinase inhibitors (CKIs). These CKIs bind to and inhibit S phase-specific CDKs (cyclin A:Cdk2 in mammalian cells, Clb5:Cdk1 in budding yeast and Cig2:Cdk1 in fission yeast). At the G1/S transition, CKI is phosphorylated and degraded, releasing the S-CDK to initiate DNA synthesis. According to our paradigm (figure 1 and table 1), bistability at the G1/S transition requires that CKI degradation be promoted by the S-CDK complex. CKI degradation is indeed promoted by CDK-mediated hyper-phosphorylation [17–19]. The abrupt degradation of Sic1 at the G1/S transition in budding yeast is usually attributed to multi-site phosphorylation of Sic1 by Cln2-dependent kinase, which is resistant to Sic1 inhibition [20]. However, given that Cln2 is degraded in S phase, phosphorylation of Sic1 by Cln2-dependent kinase alone cannot account for irreversibility of the transition. Irreversibility of the G1/S transition requires that Sic1 be hyper-phosphorylated by Clb-dependent kinases as well [21], a supposition recently confirmed by Koivomagi *et al*. [22].

Finally, the meta/anaphase transition is induced by the anaphase-promoting complex/cyclosome (APC/C). Before the transition, the APC is kept inactive by binding with its stoichiometric inhibitor, the mitotic checkpoint complex (MCC). (The MCC is a large protein complex, including Mad2 and Cdc20 among other components.) Bistability of the checkpoint mechanism requires that the MCC is inactivated by the APC. The biochemical basis of MCC inactivation during anaphase was enigmatic for a long time, until Reddy *et al*. [23] showed that disassembly of the MCC:APC complex is promoted by poly-ubiquitination of the Cdc20 subunit of the MCC, catalysed by the APC itself. Although these findings were questioned later [24], more recent publications have confirmed the conclusions of Kirschner's laboratory [25–27]. Briefly, APC promotes poly-ubiquitination of the Cdc20 molecule in the MCC complex, which leads to Cdc20 degradation, MCC disassembly and release of free APC that associates with another MCC or Cdc20.

Hence, in both cases, CKI and MCC are substrates as well as inhibitors of the very enzyme complexes they are opposing (figure 1*b*), thereby creating the potential for bistability of the molecular mechanisms and irreversibility of the associated cell cycle transitions. Furthermore, the enzymes carry out multiple modifications of their substrate/inhibitor (multi-site phosphorylation of CKIs and poly-ubiquitination of the Cdc20 subunit of the MCC).

Based on the experimental evidence summarized here, we present in this paper a general and robust mechanism of cell cycle transitions based on bistable biochemical switches. The mechanism relies on multiple modifications of an inhibitor by the enzyme it inhibits. The inhibitor strongly inhibits its first modification by the enzyme, but later it becomes a good substrate for further modifications. We characterize the properties of this mechanism by mathematical modelling, we describe a number of realizations of this mechanism in cell cycle controls, and we compare the model's properties with the observed genetic and physiological properties of cell cycle transitions.

## 3. Results

### 3.1. Substrate–inhibitor multiply modified: a general motif for bistability at cell cycle transitions

**Figure 2.**
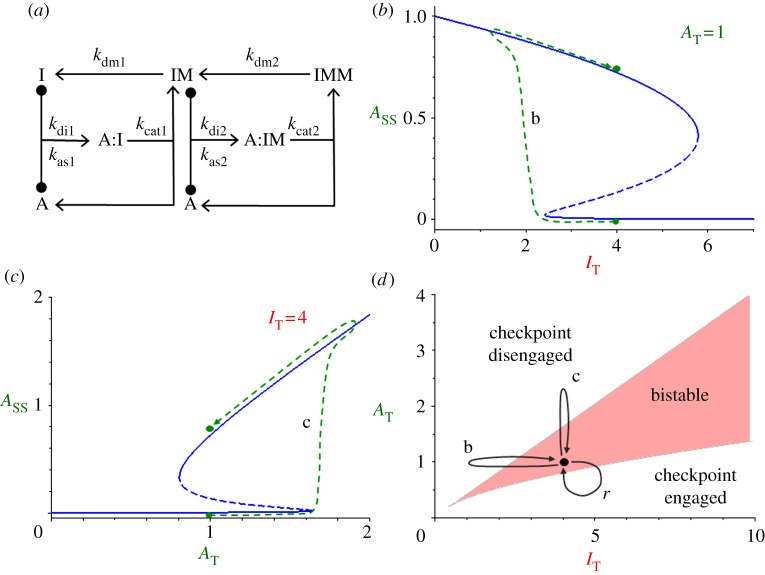
The SIMM motif. (*a*) Wiring diagram. Enzyme A binds to substrate I to form an enzyme–substrate complex, A:I, which then reacts to form a modified substrate IM plus free enzyme. Then, the same sequence of steps occurs a second time to form the doubly modified form of I. (*b*) Signal–response curve. For a fixed value of total enzyme concentration, *A*_T_ = 1, the steady-state concentration of free enzyme, *A*_ss_, is plotted as a function of total substrate concentration, *I*_T_. Path b is explained in (*d*). (*c*) Signal–response curve. For a fixed value of total substrate concentration, *I*_T_ = 4, the steady-state concentration of free enzyme, *A*_ss_, is plotted as a function of total enzyme concentration, *A*_T_. Path c is explained in (*d*). (*d*) Control plane. As a function of *A*_T_ and *I*_T_, the reaction network is bistable only within the V-shaped region. Below the V, the checkpoint is engaged (A inhibited), and above the V, the checkpoint is disengaged (A active). The black circle represents the neutral state of the checkpoint before the cell cycle transition: the reaction system is in the bistable region, in the checkpoint-engaged stable steady state. Paths b and c are possible paths for disengaging the checkpoint and triggering an irreversible cell cycle transition, as shown in (*b*,*c*). Indeed, any path that carries the control system across the upper boundary of the V and back to the neutral state will trigger an irreversible cell cycle transition. Eventually, the checkpoint must be reset to the ‘engaged’ state, which requires the system to follow a path *r* that crosses the lower boundary of the V and then returns to the neutral state.

We start with a simple model (figure 2*a*) of an inhibitor I that is multiply modified by an enzyme A, which is an activator of a cell cycle transition. IM represents ‘early’ modifications of the inhibitor, which are sluggishly accomplished and which tie up the activator in tightly bound enzyme–substrate complexes, A:I. IMM represents ‘later’ modifications, which occur quickly and (perhaps) processively. It is important that the enzyme disengage from its substrate at some point between the early and later modifications. This distributive step [28] is a consequence of our assumption that A:I is tightly bound and slowly processed, whereas A:IM is loosely bound and rapidly processed. (The model is bistable as described here even if as much as approx. 20% of the dual modification process (I → IM → IMM) occurs processively rather than distributively, as shown in the electronic supplementary material, §S1.)

We assume that the enzymatic modifications follow the Michaelis–Menten mechanism. Because enzyme A and substrate I are present in comparable concentrations, we must implement the Michaelis–Menten mechanism in terms of elementary monomolecular and bimolecular steps, obtaining a set of nonlinear ordinary differential equations (ODEs) in terms of *I* = [I], *I*_M_ = [IM], *I*_MM_ = [IMM], *C* = [A:I], and *C*_M_ = [A:IM]:3.1
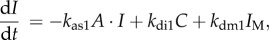
3.2
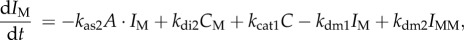
3.3
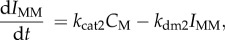
3.4
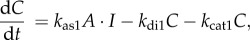
3.5
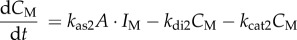
3.6
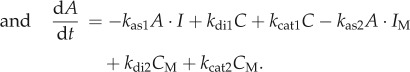


Subscripts on rate constants have the following meanings: as, association; di, dissociation; cat, catalysis; dm, de-modification; 1, first modification; 2, second modification. Note that equations (3.1)–(3.6) satisfy two conservation conditions: *A*_T_ = *A* + *C* + *C*_M_ = const. and *I*_T_ = *I* + *C* + *I*_M_ + *C*_M_ + *I*_MM_ = const. We study the behaviour of this reaction motif for the rate constant values given in table 2. These values are consistent with our assumptions that I binds very strongly to A to form a stable A:I complex, which reacts slowly to form the early-modified forms IM, and that the later-modified forms are good substrates—not good inhibitors—of A.

**Table 2.** Values of rate constants and other parameters in the models. U, concentration unit (approx. 10 nM); T, time unit (approx. 1 min).

rate constants	units	interpretation
(*a*) SIMM motif (figure 2)		
*k*_as1_ = 100, *k*_as2_ = 50	U^−1^ T^−1^	I binds more strongly to A than does IM
*k*_di1_ = 0.5, *k*_di2_ = 1	T^−1^	dissociation rate constant is same for A:I and A:IM
*k*_cat1_ = 0.5, *k*_cat2_ = 50	T^−1^	IM is a much better substrate for modification by A than is I
*k*_dm1_ = 1, *k*_dm2_ = 1	T^−1^	de-modification rate constant is same for IM and IMM
*A*_T_*, I*_T_ variable	U	total concentrations of A and I can be varied
(*b*) SIMM* motif (figure 3). Same as set (*a*), plus		
*k*_m_ = 50	U^−1^ T^−1^	rate constant for A+IM → A+IMM
*K*_m1_ = 0.01	U	Michaelis constant for first modification reactions
(*c*) Sic–Clb mechanism (figure 4*a*)		
*k*_s_ = 0.2	U T^−1^	rate of synthesis of Sic1
*k*_d_ = 0.05	T^−1^	background degradation rate for all Sic1 forms
*k*_as_b_ = 100	U^−1^ T^−1^	binding of Sic1 to Clb:Cdk1
*k*_di_b_ = 0.01	T^−1^	dissociation rate constant for Clb:Sic trimers
*k*_p_b_ = 20	U^−1^ T^−1^	rate constant for Clb-dependent phosphorylation of SicP to SicPP (followed by rapid degradation of SicPP)
*k*_dp_ = 0.5	T^−1^	dephosphorylation rate constant
*k*_cat_b_ = 0.5	T^−1^	rate constant for Clb:Sic → Clb + SicP
[ClbT] variable	U	total concentration of Clb:Cdk1 dimers
(*d*) Cln–Sic–Clb mechanism (figure 4*c*). Same as set (*c*), plus		
*k*_as_n_ = 2	U^−1^ T^−1^	binding of Sic1 to Cln:Cdk1
*k*_di_n_ = 2	T^−1^	dissociation rate constant for Cln:Sic trimers
*k*_p_n_ = 0.1	U^−1^ T^−1^	rate constant for phosphorylation of SicP by Cln-kinase
*k*_cat_n_ = 2	T^−1^	rate constant for Cln:Sic → Cln + SicP
[ClnT]	U	total concentrations of Cln:Cdk1 dimers
(*e*) MCC–APC mechanism (figure 5*a*)		
*K*_m_ = 0.01	U	Michaelis constant APC:MCC complex
*k*_u_ = 10	U^−1^ T^−1^	rate constant for ubiquitination of MCCU by APC
*k*_du_ = 5	T^−1^	rate constant for ubiquitination of MCCU by APC
*k*_cat_ = 0.5	T^−1^	rate constant for APC:MCC → MCCU + APC
*k*_a_*N*_T_ = 1	T^−1^	rate constant for activation of Mad2 by tensionless centromeres
*X*_tens_ variable	—	fraction of centromeres under tension on mitotic spindle
*k*_ssec_ = 0.1	U T^−1^	rate constant for synthesis of securin
*k*_dsec_ = 0.05	T^−1^	rate constant for degradation of securin
*k*_dsec,apc_ = 0.5	U^−1^ T^−1^	rate constant for APC-mediated degradation of securin
(*f*) CycB–MCC–APC mechanism (figure 5*d*). Same as set (*e*) (except *k*_a_*N*_T_), plus		
*k*_scyc_ = 0.01	U T^−1^	rate constant for synthesis of cyclin B
*k*_dcyc_ = 0.01	T^−1^	rate constant for degradation of cyclin B
*k*_dcyc_apc_ = 1	U^−1^ T^−1^	rate constant for APC-mediated degradation of cyclin B
*k*_an_ = 1	U^−1^ T^−1^	rate constant for activation of tensionless centromeres by CycB
*k*_in_ [CAPP] = 2	T^−1^	rate constant for inactivation of tensionless centromeres by CAPP
*k*_as_ = 100	U^−1^ T^−1^	rate constant for association of APC:MCC complex
*k*_di_ = 0.5	T^−1^	rate constant for dissociation of APC:MCC complex
*k*_a_*N*_T_ = 5	T^−1^	rate constant for activation of Mad2 by tensionless centromeres

As simple as this mechanism appears, it is a double-negative feedback loop (figure 1*b*). As long as *A*_T_ is sufficiently less than *I*_T_, most of the activator is locked up in the inactive A:I complex. However, when *A*_T_ > *I*_T_, there will be some free enzyme (*A* = *A*_T_ − *I*_T_) that can catalyse the later modification step, IM → IMM. Because the later-modified forms of I are not good inhibitors of A, the proportion of functional activator in the reaction network will increase steadily. Indeed, as shown by the signal–response curves (figure 2*b,c*), the network motif exhibits bistability for the rate constant values proposed in table 2*a*.

We assume that, when a cell is in stage *i* − 1 of the cell cycle, *I*_T_ is sufficiently greater than *A*_T_ so that the control system is locked in the state of low activity of A (checkpoint-engaged). To release the checkpoint, the mechanism must generate a signal that either decreases *I*_T_ or increases *A*_T_. When the *A*_T_/*I*_T_ ratio gets large enough, the checkpoint disengages, and the cell transitions to stage *i* of the cell cycle. (In figure 2*b*, *I*_T_ decreases at fixed *A*_T_ = 1, and the transition occurs at *I*_T_ ≈ 2.5; in figure 2*c*, *A*_T_ increases at fixed *I*_T_ = 4, and the transition occurs at *A*_T_ ≈ 1.6.)

The bistable behaviour of this reaction mechanism depends critically on the total concentrations of the inhibitor/substrate I and activator/enzyme A, as shown in figure 2*d*. Bistability is restricted to a V-shaped region in the *A*_T_, *I*_T_ plane (called a ‘control plane’ because the parameters *A*_T_ and *I*_T_ control the behaviour of the reaction mechanism). Below the V, the system is mono-stable with little active A (checkpoint engaged), and above the V, the system is mono-stable with lots of active A (checkpoint disengaged).

Bistability of the reaction motif in figure 2*a* depends on three properties of the checkpoint protein I: it is a *substrate* for enzymatic modification by A and an *inhibitor* of enzyme A as well, and I is *multiply modified* by A. Hence, we refer to figure 2*a* as a substrate–inhibitor-multiply modified (SIMM) motif. We propose that SIMM is a general motif for cell cycle checkpoints with the characteristics described in figure 1.

In figure 1*a* we stressed that a cell cycle checkpoint must be non-resettable (i.e. the signal that represents completion of stage *i*−1 must trigger the checkpoint transition, and when the signal drops to zero after the transition, the checkpoint mechanism must remain in the disengaged state). It would appear from figure 2*b,c* that our SIMM motif does not have this property, but that depends on the starting values of *I*_T_ and *A*_T_ when the signal = 0. We suggest that, when the signal = 0, the ‘neutral’ values of *I*_T_ and *A*_T_ are in the bistable region (the black dot in figure 2*d*), and the control system is in the checkpoint-engaged steady state. As the signal increases, representing progression through stage *i*−1, either *I*_T_ decreases (path b) or *A*_T_ increases (path c) until the checkpoint disengages (see the corresponding paths in figure 2*b,c*). If the signal drops to zero after the transition, the dynamical system returns to its starting point (·) on figure 2*d*, but the checkpoint is now locked in the disengaged steady state.

Sometime later in the present cell cycle or early in the next cycle, the checkpoint must be reset to the engaged state (low activity of A). This requires a separate resetting mechanism, which takes the dynamical system along path r in figure 2*d*, from the bistable starting point (·) in the disengaged state (high activity of A) across the lower switching boundary and back to the starting point in the checkpoint-engaged state.

The basic mechanism we are proposing for generating bistability by multiple modification of a checkpoint inhibitor was motivated by an earlier proposal by Kholodenko and co-workers [29,30] for bistability based on dual phosphorylation of substrates by enzymes of the MAP kinase pathway. The reaction mechanisms (figure 1*a*; fig. 1 in [30]) are similar, but the details of the analysis are different. Markevich *et al*. [30] assume that all enzymatic reactions are described by Michaelis–Menten kinetics, taking into account competition of the various substrate forms (S, SP and SPP) for the free kinase and phosphatase enzymes. Their use of Michaelis–Menten rate laws assumes that the three forms of the substrate are in great excess over the total amounts of kinase and phosphatase. Because the enzymes and substrates (A and I) operating at cell cycle transitions are present in comparable concentrations, we cannot use Michaelis–Menten rate laws but must write out the mass-action rate laws for all species in the mechanism. On the other hand, our model is a little simpler than that of Markevich *et al*. [30], because we assume that the de-modifying enzyme E (comparable to their phosphatase) operates as a mass-action catalyst (E+IM → I+E). Our mechanism is bistable even if E de-modifies IM and IMM equally rapidly. Because E is a mass-action catalyst, we can absorb its concentration into the rate constants, *k*_dm1_ and *k*_dm2_, for the de-modification reactions.

### 3.2. SIMM*: a simplified motif for bistability at cell cycle transitions

**Figure 3.**
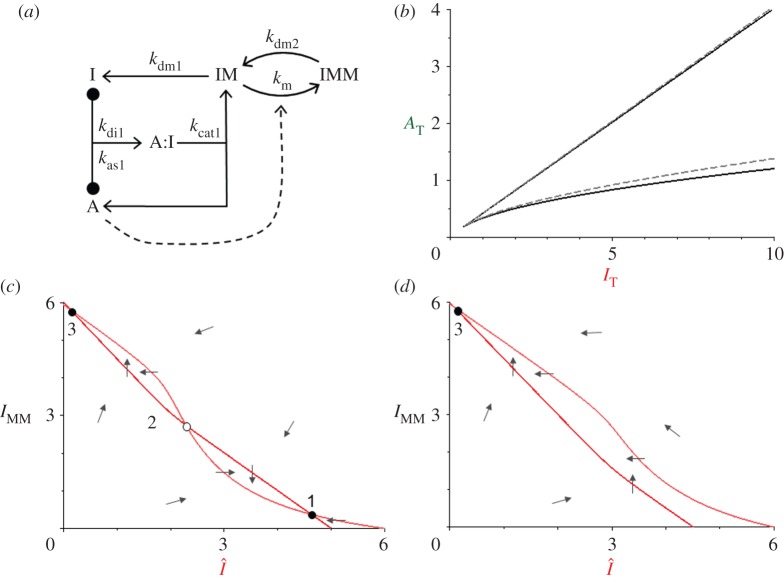
The SIMM* motif. (*a*) Wiring diagram. For the second modification of I, we replace the Michaelis–Menten mechanism by a simple mass-action rate law. (*b*) Control plane, *A*_T_ versus *I*_T_. The V-shaped bistability zone for the SIMM* motif (solid line) is almost identical to the zone for the full SIMM motif (dashed line, from figure 2*d*). (*c*) Phase plane, *I*_MM_ versus 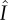, in the bistable zone (*A*_T_ = 2 and *I*_T_ = 6). There are two stable steady states—checkpoint engaged (1) and checkpoint disengaged (3)—separated by an unstable steady state (2). (*d*) Phase plane in the monostable zone (*A*_T_ = 3 and *I*_T_ = 6). There is now a single steady state: the stable checkpoint-disengaged state (3). In (*c*,*d*), the arrows indicate the direction of change of the state variables, *I*_MM_ and 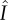, as predicted by the dynamical system, equations (3.8) and (3.9).

For the rate constant values given in table 2*a*, the Michaelis constant for the second modification, *K*_m2_ = (*k*_di2_ + *k*_cat2_)/*k*_as2_ = 1.02 U, is large compared with *K*_m1_ (0.01 U) for the first modification. (In our nomenclature, ‘*K*_m_’ refers to a classical Michaelis constant for an enzyme-catalysed reaction, and ‘U’ represents one unit of concentration of substrate.) Hence, it is reasonable to propose that the second modification can be modelled by a mass-action rate law in place of the Michaelis–Menten mechanism used so far. The simplified motif (SIMM*) in figure 3*a* is described by a smaller set of ODEs:3.1'
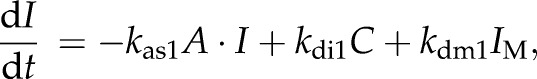
3.2'
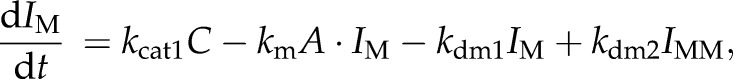
3.3'
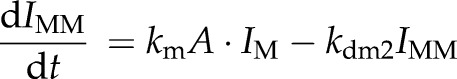
3.4'
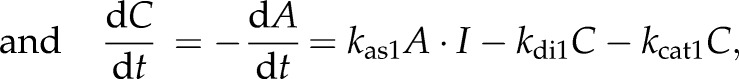
where the new rate constant is given by *k*_m_ = *k*_cat2_/*K*_m2_ = *k*_cat2_ *k*_as2_/(*k*_cat2_ + *k*_di2_) ≈ *k*_as2_. (In our nomenclature, ‘*k*_m_’ is a second-order rate constant for mass-action modification of substrate IM by enzyme A.) From the control plane for SIMM* (figure 3*b*), we see that the simplified motif reproduces almost exactly the bistability properties of the full motif (figure 2*d*).

Equations (3.1′)–(3.4′) satisfy two conservation conditions: *A*_T_ = *A* + *C* = const. and *I*_T_ = *I* + *C* + *I*_M_ + *I*_MM_ = const. As mentioned previously, because *A*_T_ and *I*_T_ are comparable, we cannot make a quasi-steady-state assumption on the enzyme–substrate complex, C, as in the derivation of the Michaelis–Menten rate law. However, as shown by Borghans *et al*. [31] and Ciliberto *et al*. [32], we may, under these circumstances, use a ‘total’ quasi-steady-state assumption (TQSSA) to simplify equations (3.1′)–(3.4′) further. Following the TQSSA, we introduce a new variable, 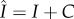, and then solve for the steady state concentration of the A:I complex in terms of *A*_T_ and 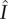: 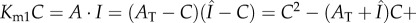 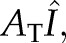 where *K*_m1_ = (*k*_di1_ + *k*_cat1_)/*k*_as1_.

Hence,3.7
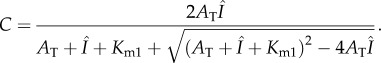


Because *A*_T_ and *K*_m1_ are constants, *C* is a function of the variable 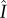. Now, equations (3.1′)–(3.4′) reduce to a pair of nonlinear ODEs for the ‘slow’ variables 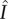 and *I*_MM_:3.8
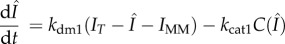
3.9
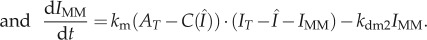


In figure 3*c*,*d* we plot the nullclines of equation (3.8) and (3.9) in the (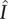, *I*_MM_) phase plane for two different values of *I*_T_. Note that these phase planes are identical to the schematic drawings in figure 1*c,d*, because 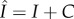 is a measure of ‘functional inhibitor’ (the two forms of the inhibitor that are functional in neutralizing activator A) and *I*_MM_ is a measure of ‘functional activator’ (the inhibitor is doubly modified only when the activator is functioning efficiently).

### 3.3. Applying SIMM* to the phosphorylation of cyclin-dependent kinase inhibitors by cyclin-dependent kinases

CKIs are an important class of CDK regulators in the cell cycle. CKIs bind to cyclin A- and cyclin B-dependent kinases to form inactive trimers (CKI:cyclin:Cdk). Because cyclin A:Cdk2 is a crucial initiator of DNA synthesis in mammalian cells, CKIs are important stabilizers of the ‘late’ G1 phase of the cell cycle (after the restriction point and before cells initiate a new round of DNA synthesis). The foremost example of a CKI is Sic1, a protein encoded by the *SIC1* gene in budding yeast [33]. The root name ‘SIC’ stands for ‘substrate and inhibitor of Cdk’. Sic1 binds to and inhibits Clb*x*:Cdk1 dimers [34], where the six B-type cyclins in budding yeast (Clb1-6) play essential roles in S/G2/M progression. Sic1 is also a target for multi-site phosphorylation by Clb*x*:Cdk1 [22]. Hence, the interactions between Sic1- and Clb-dependent kinase have the topology of a SIMM motif. Sic1 can also be phosphorylated by Cln-dependent kinases in budding yeast [17], a fact that will become important later.

**Figure 4.**
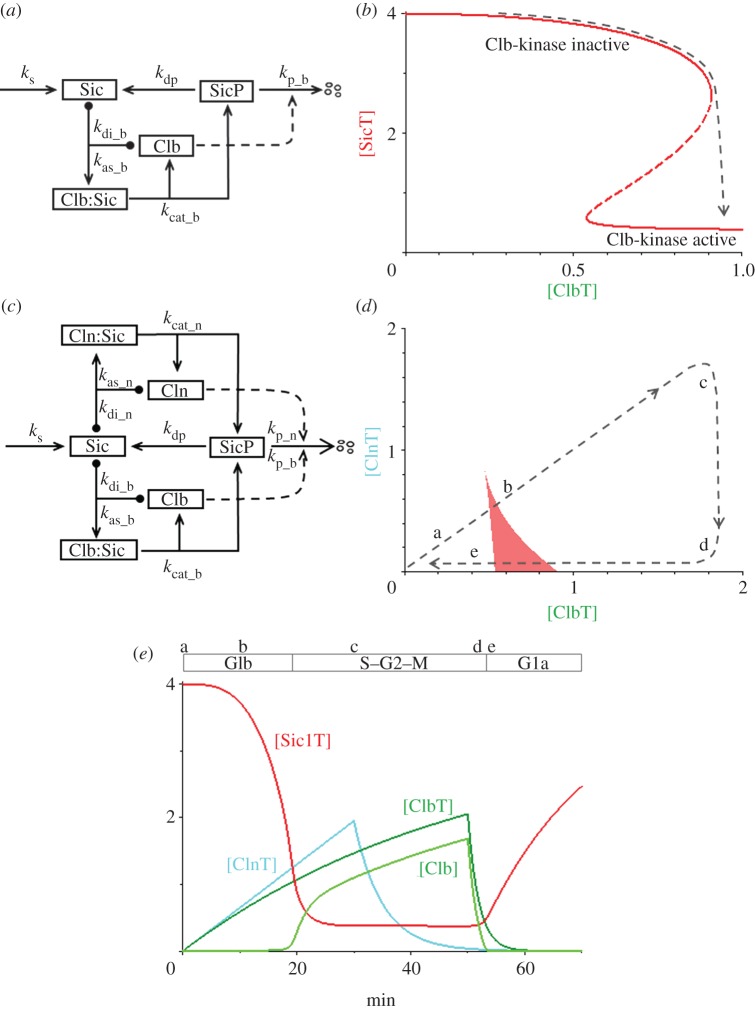
The Sic1-cyclin switch in budding yeast. (*a*) Wiring diagram for Sic1 and Clb (B-type cyclins). Sic1 is a substrate and inhibitor of Clb-dependent kinase. The second phosphorylation of Sic1 leads to its rapid degradation. (*b*) Signal–response curve. The total concentration of Sic1 in the cell (a variable) depends, in a bistable manner, on the total concentration of Clb cyclins in the cell (a parameter, in this model). The black dashed line indicates how the switch flips from the Clb-inactive state to the Clb-active state as [ClbT] increases. (*c*) Wiring diagram for Sic1, Clb and Cln (G1-type cyclins). Sic1 is a substrate—but not an effective inhibitor—of Cln-dependent kinase. (*d*) Control plane, [ClnT] versus [ClbT]. The V-shaped bistable zone is coloured red. The ‘cell cycle trajectory’ (path a–b–c–d–e) is explained in the text. (*e*) Simulation of the G1b/S transition in budding yeast, and resetting the checkpoint at cell division. Equations (3.13)–(3.16) are solved numerically for the parameter values in table 2*d*, and with additional differential equations for the synthesis and degradation of ClnT and ClbT. The synthesis terms for ClnT and ClbT are turned on at *t* = 0 (the Start transition); at *t* = 30 min, synthesis of ClnT is turned off and degradation is turned on; at *t* = 50 min (cell division), synthesis of ClbT is turned off and degradation is turned up. The G1/S transition takes place at *t* ≈ 20 min, when the cell cycle trajectory passes point b in (*d*), and there is enough active Clb-kinase to trigger DNA replication. About 50 min after Start, the cell divides and the G1 phase is re-established.

We base our new model (figure 4*a*) on the SIMM* motif in figure 3*a*, supplemented with reactions for the synthesis and degradation of Sic1. Because figure 4 refers specifically to budding yeast proteins, we change our notation somewhat: ‘CKI’ becomes ‘Sic’ and ‘cyclin:Cdk’ dimers are referred to by their cyclin subunits ‘Clb’ and ‘Cln’. With this notation, the mechanism in figure 4*a* is described by a further set of ODEs:3.10
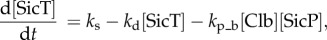
3.11
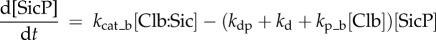
3.12
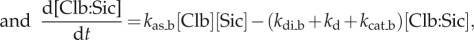
where [Sic] = [SicT] − [Clb:Sic] − [SicP] and [Clb] = [ClbT] − [Clb:Sic]. The new rate constants are *k*_s_, the rate of synthesis of Sic1, and *k*_d_, the ‘background’ rate of degradation of all forms of Sic1. We assume that the doubly phosphorylated form of Sic1 is rapidly degraded and thus lost immediately from the total pool of Sic1 subunits. The meanings and assigned values of the rate constants in equations (3.10)–(3.12) are given in table 2*c*.

Equations (3.10)–(3.12) comprise a bistable switch, as demonstrated in figure 4*b*. For low levels of total Clb-dependent kinase, the checkpoint is engaged (total Sic1 level is high). As Clb accumulates in the cell, [ClbT] increases and, when it passes the threshold, Sic1 is phosphorylated a second time and rapidly degraded (i.e. the checkpoint disengages).

### 3.4. Extending the cyclin-dependent kinase–cyclin-dependent kinase inhibitor motif to the G1/S transition in budding yeast

The model in the previous section provides a realistic mechanism for bistability of the G1/S transition in budding yeast. The two stable steady states of the bistable switch correspond to checkpoint engaged (Sic1 abundant, Clb-kinase inactive) before the transition and to checkpoint disengaged (Sic1 destroyed, Clb-kinase active) after the transition. In principle, the switch can be flipped by a signal that increases the total amount of Clb or by a signal that decreases the total amount of Sic1. In budding yeast, the signal comes from Cln-dependent kinase activity, which both upregulates synthesis of Clb proteins (by activating a transcription factor for Clb5 and Clb6) and downregulates Sic1 (by multi-phosphorylation and subsequent degradation). The Cln-type cyclins (Cln1 and Cln2) are synthesized in late G1. Cln:Cdk1 dimers are not inhibited by Sic1, but they are capable of phosphorylating Sic1. In this section, we couple the interactions of Sic1 with Cln- and Clb-dependent kinases to build a model of the G1/S transition in budding yeast.

The model (figure 4*c*) is composed of two modules: a Clb module (from the previous section), where Sic1 binds strongly to Clb:Cdk1 dimers to form inactive trimers (i.e. enzyme–substrate complexes that only slowly convert to enzyme + Sic1P); and a Cln module, where Sic1 is rapidly phosphorylated by Cln:Cdk1. The model in figure 4*c* is described by the following ODEs:3.13
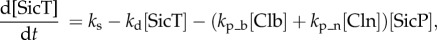
3.14
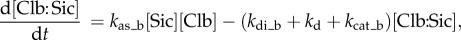
3.15
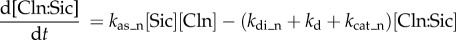
3.16
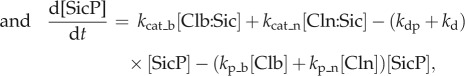
where [Sic] = [SicT] − [Clb:Sic] − [Cln:Sic] − [SicP], [Clb] = [ClbT] − [Clb:Sic] and [Cln] = [ClnT] − [Cln:Sic]. All calculations for this model are done for the rate constant values given in table 2*d*.

Figure 4*d* shows the region of bistability in the control plane spanned by [ClnT] and [ClbT]. To explain irreversibility of the G1/S transition, we plot a schematic ‘cell cycle trajectory’ (path a–b–c–d–e) on the control plane. During the second half of G1 phase (from Start to the onset of S phase), both [ClbT] and [ClnT] are increasing, as indicated by the dashed line a–c. At the beginning of this process (from a to b), the checkpoint mechanism is engaged (Sic1 abundant and Clb-kinase inactive). As the trajectory passes point b, the checkpoint mechanism disengages (Sic1 is destroyed and Clb-kinase is abundant and active). After the G1/S transition, high activity of Clb-kinase shuts off the production of Cln1 and Cln2, and [ClnT] drops along path c, d; however, along this path, [ClbT] is large enough to keep the checkpoint mechanism disengaged. Only much later in the cell cycle (during mitosis) are the Clb cyclins destroyed (path d–e–a), and the checkpoint re-engages (at point e). In figure 4*e* we simulate this scenario, to show that our model of the G1/S transition does indeed disengage and re-engage as described.

Our model confirms that both the bistability of the G1/S transition and the abruptness of Sic1 degradation are dependent on Clb cyclins [22]. These conclusions are consistent with kinetic data on Sic1 degradation in individual budding yeast cells (C. Tang 2012, personal communication).

### 3.5. Applying SIMM* to the mitotic checkpoint

The mitotic checkpoint (also known as the spindle assembly checkpoint) guards the metaphase-to-anaphase transition. As cells enter mitosis, each chromosome is fully replicated, and sister chromatids are held together by cohesin rings. Each duplicated chromosome has a centromeric region with kinetochores on each sister chromatid. During prometaphase, every chromosome must become attached by its sister kinetochores to opposite poles of the mitotic spindle. The mitotic checkpoint delays progression into anaphase (sister chromatid separation) until biorientation of the chromosomes is complete. When the last chromosome pair comes into alignment on the spindle, the checkpoint is disengaged, and separation of sister chromatids is initiated by cleavage of cohesin rings, catalysed by separase [35]. Separase is kept inactive by its stoichiometric binding partner, securin. In addition, high activity of cyclin B:Cdk1 in prometaphase and metaphase inhibits further mitotic progression [36]. Both securin and cyclin B are removed by APC-dependent poly-ubiquitination followed by proteasome-catalysed degradation [37,38]. APC requires the Cdc20 cofactor in order to ubiquitinate securin and cyclin B during mitotic progression. Therefore, APC:Cdc20 is an activator of mitotic progression that drives the cell into anaphase.

The mitotic checkpoint has three essential dynamical features: (i) even one unattached kinetochore is sufficient to keep the checkpoint engaged [39]; (ii) after the last kinetochore becomes attached, the checkpoint rapidly disengages [40]; and (iii) in anaphase, when the chromosome alignment signal drops to zero, the checkpoint does not re-engage [41–43]. These features suggest that dynamical changes in the checkpoint mechanism are crucial to proper progression through metaphase and anaphase. We have proposed that the essential features of the mitotic checkpoint derive from its bistable switching properties [44].

Unattached or mis-attached kinetochores catalyse the formation of the MCC, which binds strongly and reversibly to the APC that inhibits APC binding to free Cdc20. The APC:MCC complex includes many proteins, including Mad2, Bub3, Mad3/BubR1 and Cdc20, as well as the APC core complex [45]. As required by the SIMM motif, MCC is a substrate as well as an inhibitor of APC. MCC inactivation is an active, energy-requiring process catalysed by APC [23]. The Cdc20 subunit of the APC:MCC complex is poly-ubiquitinated by APC, which causes degradation of Cdc20 and disassembly of the complex [23,25–27,46,47]. Because Cdc20 level is not limiting during M phase, new MCCs are assembled as long as unattached kinetochores are present, giving a signal that prometaphase is still in progress. When a chromosome becomes bioriented, its centromeric region comes under tension (being pulled in opposite directions by the kinetochore attachments), and this tension silences kinetochore signalling to the MCC.

**Figure 5.**
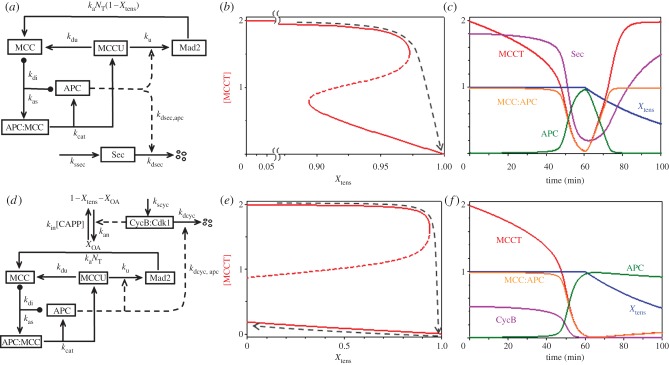
The mitotic checkpoint. (*a*) Wiring diagram. The mitotic checkpoint complex (MCC) binds to and inhibits the anaphase-promoting complex (APC). APC ubiquitinates MCC, and the ubiquitin moiety is removed by a de-ubiquitinase. Poly-ubiquitination of MCC leads to degradation of some of its components (notably Cdc20) and release of inactive checkpoint proteins (labelled by Mad2 only). Checkpoint proteins (Mad2, etc.), are reactivated by tensionless centromeres, *N*_0_ = *N*_T_(1 − *X*_tens_), leading to reassembly of MCC. Securin degradation by active APC leads to dissolution of cohesin complexes and separation of sister chromatids in early anaphase. (*b*) Signal–response curve: [MCCT], the total concentration of active MCC, versus *X*_tens_, the fraction of centromeric regions that are in tension on the mitotic spindle. The black dashed curve indicates that the mitotic checkpoint is engaged (i.e. [MCCT] large) until the last chromosome aligns on the mitotic spindle. When *X*_tens_ increases above approximately 0.97, the checkpoint disengages and APC is activated. As the cell enters anaphase, *X*_tens_ drops back to zero. (*c*) Simulation of *in vitro* release of the mitotic checkpoint. Compare with fig. 1 of Reddy *et al*. [23]. Note that when *X*_tens_ drops below approximately 0.89 the checkpoint re-engages, as predicted by the signal-response curve in (*b*). (*d*) Wiring diagram of the CycB–MCC–APC network. Cycin B-dependent kinase is required for activation of tensionless centromeres, *X*_0A_. (*e*) Signal–response curve: [MCCT] versus *X*_tens_. Now the threshold for re-engaging the checkpoint has moved to negative values of *X*_tens_, and the checkpoint remains disengaged when *X*_tens_ drops to zero in anaphase (black dashed line). (*f*) Simulation of *in vivo* release of the mitotic checkpoint. The checkpoint mechanism does not re-engage as *X*_tens_ decreases.

In summary, MCC and APC are an inhibitor and an activator locked in a double-negative feedback loop [48], as required for a SIMM motif (figure 5*a*). From the reaction mechanism in figure 5*a*, it is obvious that the total concentration of APC, [APCT] = [APC] + [APC:MCC], is constant and that the total concentration of Mad2, [Mad2T] = [Mad2] + [MCCU] + [APC:MCC] + [MCC], is also constant. It is convenient to define a new variable [MCCT] = [MCC] + [APC:MCC] + [MCCU] = total concentration of mitotic checkpoint complexes. Using these definitions, we can write a pair of nonlinear ODEs that describe our model of the interactions between MCC and APC:3.17
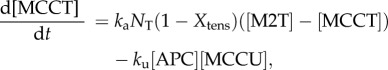
3.18
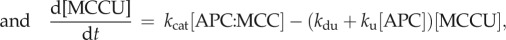
and
3.19
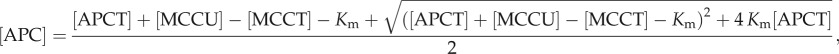


where [APC:MCC] = [APCT] − [APC] and *K*_m_ = (*k*_di_ + *k*_cat_)/*k*_as_. Equation (3.19) is derived, according to the TQSSA, by solving the following algebraic equation for [APC]: 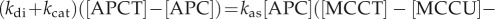 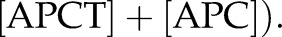

An equation for APC-mediated degradation of securin is added as an indicator of the metaphase-to-anaphase transition:3.20
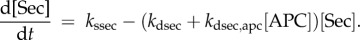


The parameters *N*_T_ and *X*_tens_ in equation (3.17) are the total number of chromosomes in the cell and the fraction of centromeres that are under tension because their two kinetochores are attached to opposite poles of the mitotic spindle. We assume that Cdc20 is present in excess during mitosis and MCC formation is limited by Mad2 (or any other MCC subunit). However, as long as APC is occupied by MCC it cannot ubiquitinate its substrates (Securin and cyclin B), whose degradation is required for anaphase progression. During prometaphase, *X*_tens_ increases from 0 to 1. The meanings and assigned values of the other constants in equations (3.17)–(3.20) are given in table 2*e*.

At the beginning of prometaphase, when *X*_tens_ = 0, the control system is trapped in a stable steady state with lots of active MCC, and most of the APC is bound up in inactive APC:MCC complexes. As chromosomes come into alignment on the spindle, the checkpoint remains engaged until *X*_tens_ gets very close to 1 (figure 5*b*). For the parameter set in table 2*e*, the mitotic checkpoint does not disengage until *X*_tens_ > *X*_disengage_ ≈ 0.97.

The evident ‘sensitivity’ of the mitotic checkpoint to even a single unaligned chromosome is not a freak consequence of our choice of rate constants in table 2*e*. In the electronic supplementary material, §S2 we use two-parameter bifurcation diagrams to show that 0.95 < *X*_disengage_ < 1 over a broad range of values of the rate constant in equations (3.17)–(3.19).

In figure 5*c* we show a simulation of how fast the MCC disengages after the last centromere comes under tension. At first there is a considerable delay, as the excess supply of ‘functional’ MCC, [MCC] + [APC:MCC], is slowly ubiquitinated by the APC. After approximately 45 min, in our simulation, total functional MCC becomes comparable with total APC, and there is now a large enough concentration of free APC to carry out the second stage of ubiquitination, which is an accelerating, autocatalytic process in our simulations and in experimental measurements [23]. *In vitro* experiments on the processing of APC:MCC complexes show a long delay (approx. 30 min) before anaphase substrates (securin and cyclin B) start to be destroyed [23], as predicted by our model. However, the long delay in figure 5*c* is not consistent with *in vivo* observations [40], where anaphase substrates are degraded within minutes of lifting the mitotic checkpoint. This disagreement between model and experiment suggests that some other processes are at work *in vivo*. The long delay in figure 5*c* is a result of the rapid rate of de-ubiquitination of MCCU, which is a necessary requirement for sensitivity of the mitotic checkpoint. During the lag phase, as functional MCC is ubiquitinated by APC, the de-ubiquitinating enzyme (USP44 in mammalian cells) is rapidly recycling MCCU to MCC. Hence, it takes a long time to poly-ubiquitinate MCC and destroy the inhibitor. Seeing it this way, we suggest that *in vivo* there are mechanisms that downregulate the activity of the de-ubiquitinase as the mitotic checkpoint disengages, so that functional MCC can be more rapidly destroyed.

### 3.6. Extending the anaphase-promoting complex–mitotic checkpoint complex motif to account for irreversibility of the metaphase-to-anaphase transition

In §3.5 we showed how a SIMM* motif might account for ‘sensitivity’ of the mitotic checkpoint and how it might be modified to account for ‘fast release’. In this section, we describe an extension of the model to account for ‘irreversibility’ of the metaphase-to-anaphase transition.

The signal–response diagram in figure 5*b* shows that the mitotic checkpoint disengages when the fraction of bioriented chromosomes gets close to 1. After the transition, centromeric cohesins are destroyed and sister chromatids move towards opposite poles of the spindle. Because their kinetochores are no longer silenced by centromeric tension, we might expect the mitotic checkpoint signal to go back to a large value. If so, then figure 5*b* predicts that the checkpoint would re-engage when *X*_tens_ drops below approximately 0.89. To account for irreversibility of the metaphase-to-anaphase transition, we must introduce additional regulatory steps to prevent re-activation of the MCC during anaphase.

Reactivation of the mitotic checkpoint in anaphase does not happen during normal mitotic progression because cyclin B:Cdk1 activity appears to be essential for checkpoint signalling [49]. Cyclin B:Cdk1 activity is high in prometaphase when tensionless kinetochores are signalling. But cyclin B is degraded by the APC during metaphase, so the mitotic checkpoint cannot be reactivated during anaphase. The non-resettable character of the mitotic checkpoint depends on cyclin B degradation by APC [42,43]. Therefore, mitotic checkpoint control is based on one activator (APC) and two inhibitors (cyclin B:Cdk1 and MCC). In addition to the SIMM motif involving APC and MCC, APC and cyclin B are engaged in a double-negative feedback loop: (i) cyclin B:Cdk1 indirectly inactivates APC by promoting MCC assembly at tensionless kinetochores; and (ii) cyclin B becomes a substrate of APC-mediated ubiquitination and degradation, once the checkpoint is silenced (figure 5*d*).

The extended SIMM motif (figure 5*d*) is governed by the following ODEs:3.21
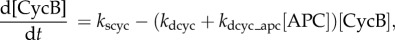
3.22
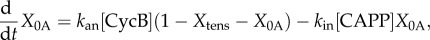
3.23
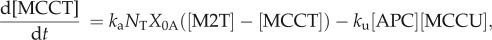
3.24
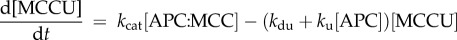
3.25
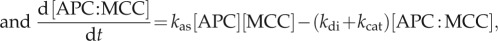
where [APC] = [APCT] − [APC:MCC] and [MCC] = [MCCT] − [APC:MCC] − [MCCU]; and *X*_0A_ indicates the fraction of centromeres without tension that are activating the MCC. Given the parameter values in table 2*f*, this model predicts that for *X*_tens_ = 0 the mitotic checkpoint has two stable steady states (figure 5*e*): ‘engaged’ ([MCCT] high, [CycB] high, [APC] low) and ‘disengaged’ ([MCCT] low, [CycB] low, [APC] high). The checkpoint starts in the engaged state in prometaphase with *X*_tens_ = 0 and ends up in the disengaged state in telophase with *X*_tens_ = 0.

One might well ask, if the mitotic checkpoint is inactive in the final state of the one-way switch (figure 5*e*), how does it get activated again in the next cell cycle? During G1 phase of the next cycle, the mitotic activity of APC (which is dependent on an auxiliary protein, Cdc20) is lost, because the Cdk1/CycB-dependent production of Cdc20 is terminated, Cdc20 protein is rapidly degraded and Cdk1/CycB-dependent activatory phosphorylations of APC are lost. The inactivation of APC/Cdc20 allows reactivation of the mitotic checkpoint in G1 phase of the next cycle.

In the electronic supplementary material, §S3 we investigate the robustness of this model of the mitotic checkpoint with respect to the dual requirements of sensitivity and irreversibility. We show that the region of parameter space where equations (3.21)–(3.25) exhibit both properties is quite restricted, compared with the robustness of our earlier model of the mitotic checkpoint [44]. The major difference between the present model and that of He *et al*. [44] is that the counter-acting protein phosphatase (CAPP in equation (3.22)) is regulated by CycB-kinase in [44], but not here. Hence, we may conclude that the simple model considered here is sufficient to account for sensitivity and irreversibility of the mitotic checkpoint. However, the mechanism is not very robust and release from the checkpoint is rather sluggish. The robustness issue can be solved by additional regulatory signals on the counter-acting protein phosphatase, and the sluggishness issue can be solved by additional regulatory signals on the counter-acting de-ubiquitinase enzyme.

## 4. Discussion

### 4.1. An underlying design principle for eukaryotic cell cycle transitions

Cell cycle transitions are impelled by specific activators: Cdks drive the transitions from G1 to S and from G2 to M, whereas the APC drives exit from mitosis back to G1. In the pre-transition state, the activator is shut down by an inhibitor. The inhibitors and activators functioning at specific cell cycle transitions are listed in table 1. In each case, prior to the transition, the inhibitor binds to the activator and inactivates it, either stoichiometrically (by forming an inactive heteromeric complex) or catalytically (by covalently modifying the activator). After the transition, the activator inactivates the inhibitor, either directly or indirectly. This double-negative feedback loop between activator and inhibitor is the basis of a bistable switch that makes the cell cycle transition a unidirectional (irreversible) change.

In this work, we analyse the case where the activator inactivates the inhibitor by direct modification. In this case, the inhibitor is both a stoichiometric inhibitor and a substrate of the activator. This relationship between inhibitor and activator is exhibited in the cell cycle at the G1/S transition (activator = Cdk and inhibitor = CKI) and at the meta-to-anaphase transition (activator = APC, inhibitor = MCC). We show that the antagonism between inhibitor and activator creates a bistable switch if (i) the inhibitor is modified by the activator more than once (multi-site modification) in separate collisions (distributive mechanism) and (ii) the modifications of the inhibitor are reversed by counter-acting reactions.

Both of these requirements are satisfied at the G1/S and meta/anaphase transitions. CKIs get phosphorylated at multiple sites, while the Cdc20 component of the MCC gets poly-ubiquitinated. CKI phosphorylation and MCC ubiquitination are reversed by phosphatase and de-ubiquitinase enzymes, respectively. The bistable switch is characterized by two sharp thresholds. The thresholds are sensitively dependent on upstream regulators and the counter-acting enzymes (the phosphatase or de-ubiquitinase) in the network.

### 4.2. Application of this principle to other cell cycle transitions and checkpoint mechanisms

Our proposed network can be easily extended to the Start transition in yeasts (and the analogous restriction point in mammalian cells). The activator of the Start transition is a transcription factor, SBF, that drives expression of G1 cyclins, Cln1 and Cln2. (In mammalian cells, the analogous components are the E2F family of transcription factors and the family of E-type cyclins.) The inhibitor is Whi5 (the RB in mammalian cells), which binds to and inactivates SBF. In this case, inactivation of the inhibitor by the activator is indirect: the SBF-induced, G1 cyclin-dependent kinases phosphorylate and inactivate the inhibitor, Whi5. It is noteworthy that Whi5 is multiply phosphorylated by Cdk1:Cln complexes. Hence, the mechanism of the Start transition bears many similarities to our SIMM motif.

**Figure 6.**
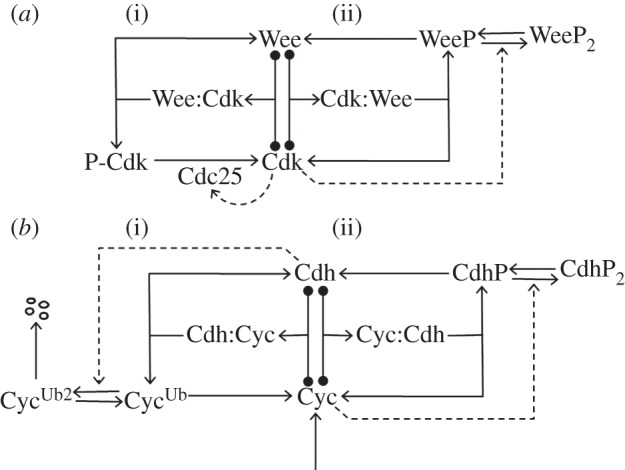
Wiring diagrams for other cell cycle transitions. (*a*) The G2/M transition. Wee1 is a protein kinase that phosphorylates and inactivates Cdk1 (i). Simultaneously, active Cdk1 (complexed with cyclin B) phosphorylates Wee1 on multiple sites in a typical SIMM motif (ii), where Wee1 functions as a stoichiometric inhibitor of Cdk1 as well as a substrate. Cdc25, the phosphatase that dephosphorylates P-Cdk1, is activated by multi-site phosphorylation catalysed by active Cdk1. (*b*) Mitotic exit. Cdh1 (in combination with the APC) stabilizes G1 phase of the cell cycle by poly-ubiquitinating cyclin B (i). Simultaneously, active Cdk1:CycB phosphorylates Cdh1 on multiple sites (ii). Both sides of the interaction have SIMM topology, but it is not known if either of the ‘substrates’ serves also as a ‘stoichiometric inhibitor’ of the enzyme.

The G2/M transition in eukaryotes is promoted by Cdk1:CycB complexes, which are kept inactive in G2 phase by inhibitory phosphorylation of Cdk1 subunits, catalysed by Wee1-like kinases (figure 6*a*). Wee1 (the inhibitor of our scheme) gets phosphorylated at multiple sites by Cdk1:CycB (the activator), and multiply phosphorylated Wee1 is less active [12]. This case differs from our SIMM motif in that the inhibitor inactivates the activator by covalent modification after complex formation. But stoichiometric inhibition of Cdk1:CycB by Wee1 is also at play here, as indicated by the phenotype of fission yeast cells carrying mutant Cdk1 subunits that cannot be phosphorylated by Wee1. These mutant cells (*cdk1^AF^*) are poorly viable, whereas fission yeast cells lacking both inhibitory kinases (*wee1^ts^ mik1*Δ**) are totally inviable. The double mutant cells undergo mitotic catastrophe (i.e. they enter mitosis before they complete DNA replication) and die, whereas some mutant cells carrying non-phosphorylable Cdk1 subunits survive, presumably because the inhibitory kinases delay the G2/M transition solely on the basis of stoichiometric (rather than catalytic) inhibition of Cdk1:CycB. The advantage of enzymatic inhibition of the activator at the G2/M transition is that much less inhibitor (Wee1) is required to stabilize the G2 state. The disadvantage is that the mechanism requires a second enzyme (Cdc25 phosphatase) to reverse this modification. Interestingly, Cdc25 is also phosphorylated by Cdk1:CycB at multiple sites; these phosphorylations are activatory, creating a positive feedback loop between Cdk1 and Cdc25.

Finally, in G1 phase of the budding yeast cell cycle, the APC combines with a different auxiliary protein, Cdh1, to serve as an inhibitor of the G1/S transition and an activator of the mitotic exit transition. APC:Cdh1 interacts with Cdk1:CycB (an activator of the G1/S transition, and an inhibitor of mitotic exit). APC:Cdh1 inhibits Cdk1:CycB by poly-ubiquitinating CycB, which induces CycB degradation by the proteasome. Cdk1:CycB inhibits APC/Cdh1 by phosphorylating the Cdh1 subunit on multiple sites. Hence, this regulatory motif consists of two SIMMs back to back (figure 6*b*). In this topology, bistability does not require that either substrate be a stoichiometric inhibitor of the enzyme to which it binds. However, bistability does require multiplicity of one or both of the catalytic processes (poly-ubiquitination and/or multiple phosphorylation).

As simple, robust mechanisms for bistable, irreversible transitions, SIMM motifs may underlie other cellular ‘decision-making’ processes in signalling networks and developmental pathways.

## 5. Material and methods

### 5.1. Mathematical modelling and simulation

Each cell cycle transition mechanism is represented as a wiring diagram, which is then converted into a set of rate equations (nonlinear ODEs) by the law of mass action. The ODEs are solved numerically using a software package called XPPAUT, which is freely available from http://www.math.pitt.edu/~bard/xpp/xpp.html. Before doing any simulations, we must assign numerical values to all the rate constants introduced by the law of mass action. We make these assignments intuitively, according to the following principles:

— The rate constants should be ‘reasonable’ numbers. For example, the rate of association of two proteins to form a complex should not be faster than the diffusion-limited rate for second-order chemical reactions, which (for proteins in cytoplasm) is approximately 10^9^ l mol^−1^ s^−1^, or approximately 600 U^−1^ min^−1^ in our units. The degradation rates of proteins such as cyclin and CKI should be consistent with observed rates (half-life∼min), and their rates of synthesis should be consistent with intracellular concentrations in the 10–100 nM range.
— The rate constant values should give signal–response curves (one-parameter bifurcation diagrams) and control planes (two-parameter bifurcation diagrams) that are consistent with the observed dynamic properties of cell cycle checkpoints (bistability, irreversibility and fast release).

In addition to providing algorithms for numerical solutions of ODEs, XPPAUT provides a suite of tools for the analysis of dynamical systems, including phase plane portraits and bifurcation analysis. We use these tools to illustrate the dynamical properties of checkpoint mechanisms. For example, figure 3*c* and 3*d* are phase plane portraits of the SIMM* motif, the ‘signal–response curves’ in figures 2, 4 and 5 are one-parameter bifurcation diagrams, and the ‘control planes’ in figures 2, 3 and 4 are two-parameter bifurcation diagrams. Interested readers should consult the electronic supplementary material, §S4 for a simple introduction to these tools.

All figures in this paper can be reproduced in XPPAUT using the ‘.ode’ files provided in the electronic supplementary material, §S5.

## 6. Acknowledgements

This work was supported in part by the National Science Foundation's award number DBI-0904340 (to A.V.), by the National Institutes of Health's award no. 5R01GM078989-06 (to J.J.T.), and by the BBSRC and European Community's Seventh Framework Programmes UniCellSys/201142 and MitoSys/241548 (to B.N.).

## Supplementary Material

Supplementary Material
