# Supplementary material for: Molecular mechanisms creating bistable switches at cell cycle transitions
Source: Open Biol. 2013 Mar;3(3):120179. doi: 10.1098/rsob.120179 (PMC3718337; doi:10.1098/rsob.120179)
Supplement: Supplementary Material [file rsob120179-s1.pdf]

Supplementary Material

Molecular Mechanisms Creating Bistable Switches at Cell Cycle Transitions

by

Anael Verdugo[a,b,c], P.K. Vinod[a,c], John J. Tyson[b], Bela Novak[a,*]

Supplement 1. Distributive and Processive Pathways for Multiple Modification

In Fig. S1A we modify Fig. 2A to allow the two modifications of I to proceed by either a processive pathway, A:I → A:IM* → A:IM → A + IMM, or a distributive pathway, A:I → A:IM* → A + IM → A:IM → A + IMM. Here we analyze the bistability properties of this mechanism as we vary the rate k_{proc} of the processive step A:IM* → A:IM.

The differential equations governing the wiring diagram in Fig. S1A are

$$\frac{dI}{dt} = -k_{\text{as1}}A \cdot I + k_{\text{di1}}C + k_{\text{dm1}}I_{\text{M}} \tag{S1.1}$$

$$\frac{dI_{\text{M}}}{dt} = -k_{\text{as2}}A \cdot I_{\text{M}} + k_{\text{di2}}C_{\text{M}} - k_{\text{dm1}}I_{\text{M}} + k_{\text{dm2}}I_{\text{MM}} + k_{\text{dist}}C_{\text{M}}^{*} \tag{S1.2}$$

$$\frac{dI_{\text{MM}}}{dt} = k_{\text{cat2}}C_{\text{M}} - k_{\text{dm2}}I_{\text{MM}} \tag{S1.3}$$

$$\frac{dC}{dt} = k_{\text{as1}}A \cdot I - k_{\text{di1}}C - k_{\text{cat1}}C \tag{S1.4}$$

$$\frac{dC_{\text{M}}}{dt} = k_{\text{as2}}A \cdot I_{\text{M}} - k_{\text{di2}}C_{\text{M}} - k_{\text{cat2}}C_{\text{M}} + k_{\text{proc}}C_{\text{M}}^{*} \tag{S1.5}$$

$$\frac{dA}{dt} = -k_{\text{as1}}A \cdot I + k_{\text{di1}}C + k_{\text{dist}}C_{\text{M}}^{*} - k_{\text{as2}}A \cdot I_{\text{M}} + k_{\text{di2}}C_{\text{M}} + k_{\text{cat2}}C_{\text{M}} \tag{S1.6}$$

$$\frac{dC_{\text{M}}^{*}}{dt} = k_{\text{cat1}}C - k_{\text{dist}}C_{\text{M}}^{*} - k_{\text{proc}}C_{\text{M}}^{*} \tag{S1.7}$$

subject to two conservation conditions: $A_{\text{T}} = A + C + C_{\text{M}} + C_{\text{M}}{*}$ = constant and $I_{\text{T}} = I + I_{\text{M}} + I_{\text{MM}} + C + C_{\text{M}} + C_{\text{M}}{*}$ = constant. Notice that, if $k_{\text{proc}} = 0$, then Equations (S1.1)-(S1.6) become identical to Equations (1)-(6) in the main text, when we substitute $k_{\text{dist}}\, C_{\text{M}}{*} = k_{\text{cat1}}\, C$, as implied by (S1.7). We expect that $k_{\text{dist}} >> k_{\text{cat1}}$ (i.e., the catalysis step is slow compared to the release of IM from the enzyme-product complex), so we choose $k_{\text{dist}} = 20\ \text{T}^{-1}$ as a reasonable value.

In Fig. S1B we plot the two-parameter bifurcation diagram for Equations (S1.1)-(S1.7) with I_T and k_{proc} as the primary and secondary bifurcation parameters, and $A_T = 1$ (fixed). We see that, for $k_{proc} = 0$, the saddle-node bifurcations occur at $I_T = 2.0$ and 5.7 (not too different from the bifurcation points in Fig. 2B). As k_{proc} increases, the bistable interval $I_{SN1} < I_T < I_{SN2}$ shrinks, but bistability is not lost completely until $k_{proc} \approx k_{dist}$. Certainly, as long as the processive pathway accounts for less than ~20% of the fate of the A:IM* complex, the SIMM motif shows robust bistability.

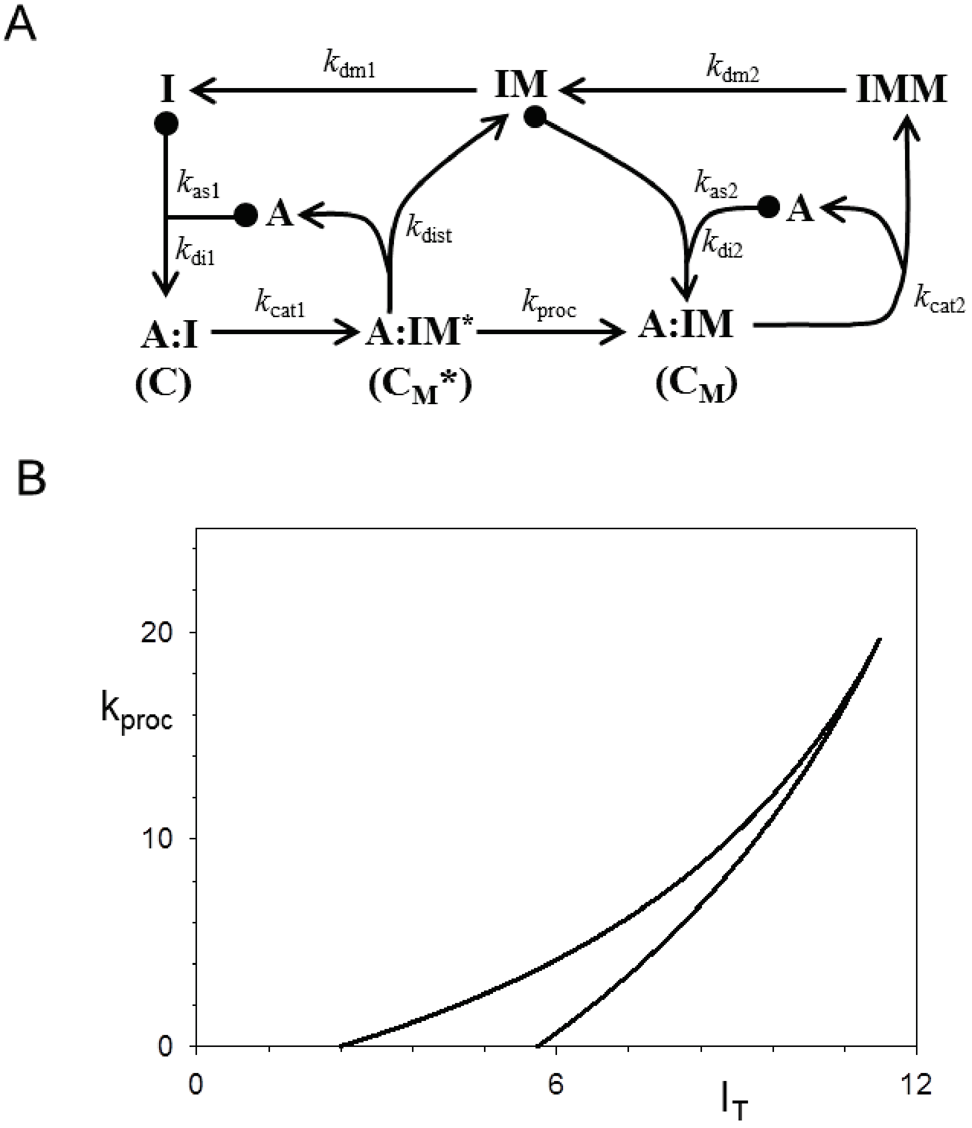

Supplementary Figure S1. Distributive and processive pathways for multiple modification of inhibitor I by activator A. (A) Wiring diagram. (B) Control plane.

Supplement 2. Sensitivity of the Mitotic Checkpoint

To function properly the mitotic checkpoint must be sensitive to even one unaligned chromosome out of a total of (say) 20 or more chromosomes. This level of sensitivity demands that the MCC be actively engaged even for values of X_{tens} (see Equation 17) very close to 1. This requirement means that the "threshold for MCC disengagement" (see Fig. 5B) must lie between (say) 0.95 and 1. For the parameter values (Table 2E) used to compute Fig. 5B, this requirement is satisfied ($X_{\text{disengage}} \approx 0.97$); but how robust is this behavior to changes in the kinetic parameters of the model?

To answer this question, it is convenient to cast Equations (17)-(19) in dimensionless form, in order to minimize the number of independent parameters that determine the locations of the saddle-node bifurcation points. To this end, we define the following dimensionless variables:

$$C_{\text{APC}} = \frac{[\text{APC}]}{[\text{APCT}]}, C_{\text{MCCT}} = \frac{[\text{MCCT}]}{[\text{APCT}]}, C_{\text{MCCU}} = \frac{[\text{MCCU}]}{[\text{APCT}]}, C_{\text{APCMCC}} = \frac{[\text{APC:MCC}]}{[\text{APCT}]}. \quad \text{(S2.1)}$$

In these terms, we rewrite Equations (17)-(19) as follows:

$$\frac{1}{k_{\text{cat}}}\frac{d}{dt}C_{\text{MCCT}} = \gamma(1 - X_{\text{tens}})(\Psi - C_{\text{MCCT}}) - \delta C_{\text{APC}}C_{\text{MCCU}} \quad \text{(S2.2)}$$

$$\frac{1}{k_{\text{cat}}}\frac{d}{dt}C_{\text{MCCU}} = C_{\text{APCMCC}} - \varphi C_{\text{MCCU}} - \delta C_{\text{APC}}C_{\text{MCCU}} \quad \text{(S2.3)}$$

$$\frac{1}{k_{\text{di}}+k_{\text{cat}}}\frac{d}{dt}C_{\text{APCMCC}} = \kappa(1 - C_{\text{APCMCC}})(C_{\text{MCCT}} - C_{\text{APCMCC}} - C_{\text{MCCU}}) - C_{\text{APCMCC}} \quad \text{(S2.4)}$$

We have retained the differential equation (S2.4) for C_{APCMCC}, instead of using the TQSSA. The 5 dimensionless parameters in Equations (S2.2)-(S2.4) are defined as follows:

$$\gamma = \frac{k_{\text{a}}N_{\text{T}}}{k_{\text{cat}}} = 2,\ \Psi = \frac{[\text{M2T}]}{[\text{APCT}]} = 2,\ \delta = \frac{k_{\text{u}}[\text{APCT}]}{k_{\text{cat}}} = 20,\ \varphi = \frac{k_{\text{du}}}{k_{\text{cat}}} = 10,\ \kappa = \frac{[\text{APCT}]}{K_{\text{m}}} = 100. \quad \text{(S2.5)}$$

The numerical values assigned to these parameters come from Table 2E.

In Fig. S2 we plot 5 two-parameter bifurcation diagrams, which indicate how the threshold for MCC disengagement, $X_{\text{disengage}}$, depends on the 5 dimensionless parameters in Equations (S2.2)-(2.4). These two-parameter bifurcation diagrams determine the bounds on the 5 parameters that must be respected in order that $0.95 < X_{\text{disengage}} < 1$. These bounds are (approximately):

$$\gamma > 1.8,\ \Psi > 1.9,\ 0 < \delta < 23,\ \varphi > 8.7,\ \kappa > 82. \quad \text{(S2.6)}$$

Because these bounds are not very restrictive, it would appear that "sensitivity" is a robust property of our model of the mitotic checkpoint. However, Fig. S2 also indicates that the threshold for re-engagement of the mitotic checkpoint is only slightly lower than $X_{\text{disengage}}$. Hence, this model of the mitotic checkpoint is not "irreversible", and we fix that problem in the next Supplement.

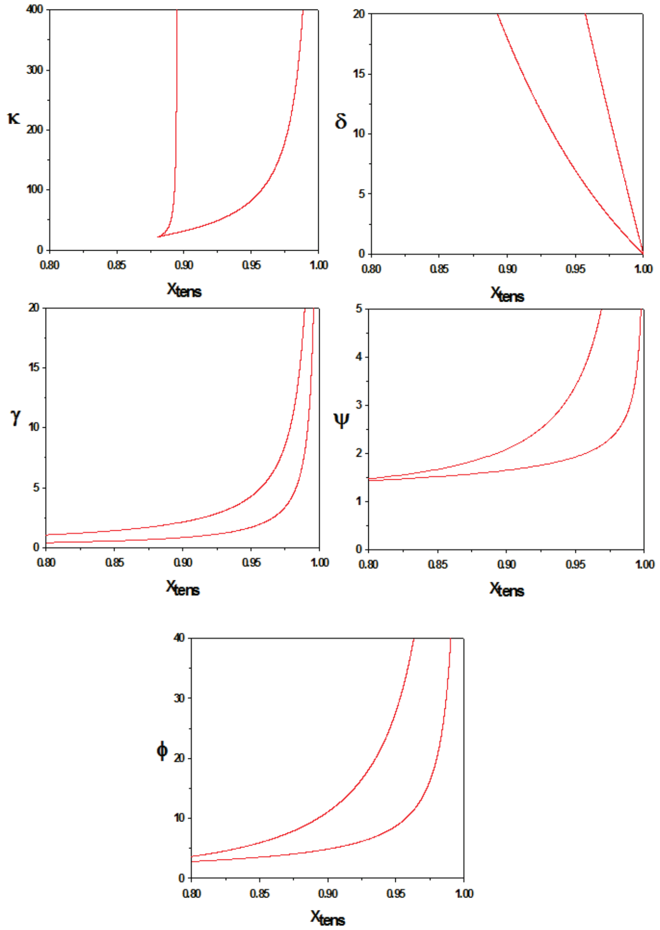

Supplementary Figure S2. Two-parameter bifurcation diagrams for the mitotic checkpoint model in Fig. 5D and defined by Equations (S2.2)-(S2.4). For the dimensionless parameter indicated on the vertical axis in each panel, the checkpoint control mechanism is bistable in the region between the two red curves. The left- and right curves plot $X_{\text{re-sengage}}$ and $X_{\text{disengage}}$, respectively, as functions of the parameter. "Sensitivity" of the mitotic checkpoint requires (roughly) that $0.95 < X_{\text{disengage}} < 1.0$, which puts bounds on the parameters, see Equation (S2.6).

Supplement 3. Robust Irreversibility of the Mitotic Checkpoint

Irreversibility of the mitotic checkpoint means that the control system is bistable for $X_{\text{re-engage}} < X_{\text{tens}} < X_{\text{disengage}}$, with $0.95 < X_{\text{disengage}} < 1$ (so that the checkpoint remains engaged even if as few as 5% of the cell's chromosomes are unaligned) and $X_{\text{re-engage}} < 0$ (so that the checkpoint does not re-engage when X_{tens} drops to 0 in anaphase). In the text and in Supplement 2, we show that the SIMM model of the mitotic checkpoint, Equations (17)-(19) is not irreversible, but the extended model, Equations (21)-(25), is irreversible. Does the extended model exhibit robust irreversibility? That is, are the requirements for irreversibility satisfied over appreciable ranges of the other thirteen parameters in Equations (21)-(25)?

As in Supplement 2, we minimize the number of independent parameters by rewriting the differential equations in terms of dimensionless variables, defined in Equations (S2.1) and:

$$C_{\text{CycB}} = \frac{k_{\text{dcyc}}[\text{CycB}]}{k_{\text{scyc}}}. \tag{S3.1}$$

Equations (21)-(25) become:

$$\frac{1}{k_{\text{dcyc}}}\frac{d}{dt}C_{\text{CycB}} = 1 - (1 + \beta C_{\text{APC}})C_{\text{CycB}} \tag{S3.2}$$

$$\frac{1}{k_{\text{in}}[\text{CAPP}]}\frac{d}{dt}X_{0\text{A}} = \Theta C_{\text{CycB}}(1 - X_{\text{tens}} - X_{0\text{A}}) - X_{0\text{A}} \tag{S3.3}$$

$$\frac{1}{k_{\text{cat}}}\frac{d}{dt}C_{\text{MCCT}} = \gamma X_{0\text{A}}(\Psi - C_{\text{MCCT}}) - \delta C_{\text{APC}}C_{\text{MCCU}} \tag{S3.4}$$

$$\frac{1}{k_{\text{cat}}}\frac{d}{dt}C_{\text{MCCU}} = C_{\text{APCMCC}} - \varphi C_{\text{MCCU}} - \delta C_{\text{APC}}C_{\text{MCCU}} \tag{S3.5}$$

$$\frac{1}{k_{\text{di}}+k_{\text{cat}}}\frac{d}{dt}C_{\text{APCMCC}} = \kappa(1 - C_{\text{APCMCC}})(C_{\text{MCCT}} - C_{\text{APCMCC}} - C_{\text{MCCU}}) - C_{\text{APCMCC}} \tag{S3.6}$$

The 7 dimensionless parameters in these equations are defined in Table S1.

In Figure S3 we plot 7 two-parameter bifurcation diagrams, which indicate how the two saddle-node bifurcation points, $X_{\text{re-engage}}$ and $X_{\text{disengage}}$, depend on the 7 dimensionless parameters in Table S1. These two-parameter bifurcation diagrams determine the bounds on the 7 parameters that must be respected in order to satisfy the requirements for checkpoint irreversibility: $X_{\text{re-engage}} < 0$ and $0.95 < X_{\text{disengage}} < 1$. These bounds are summarized in Table S1

and compared to the bounds determined for the model of He et al. (2011). The model presented here is considerably less robust than the model of He et al. The difference can be attributed to the fact that the counter-acting protein phosphatase (CAPP) is regulated by CycB-kinase in He et al. but not here.

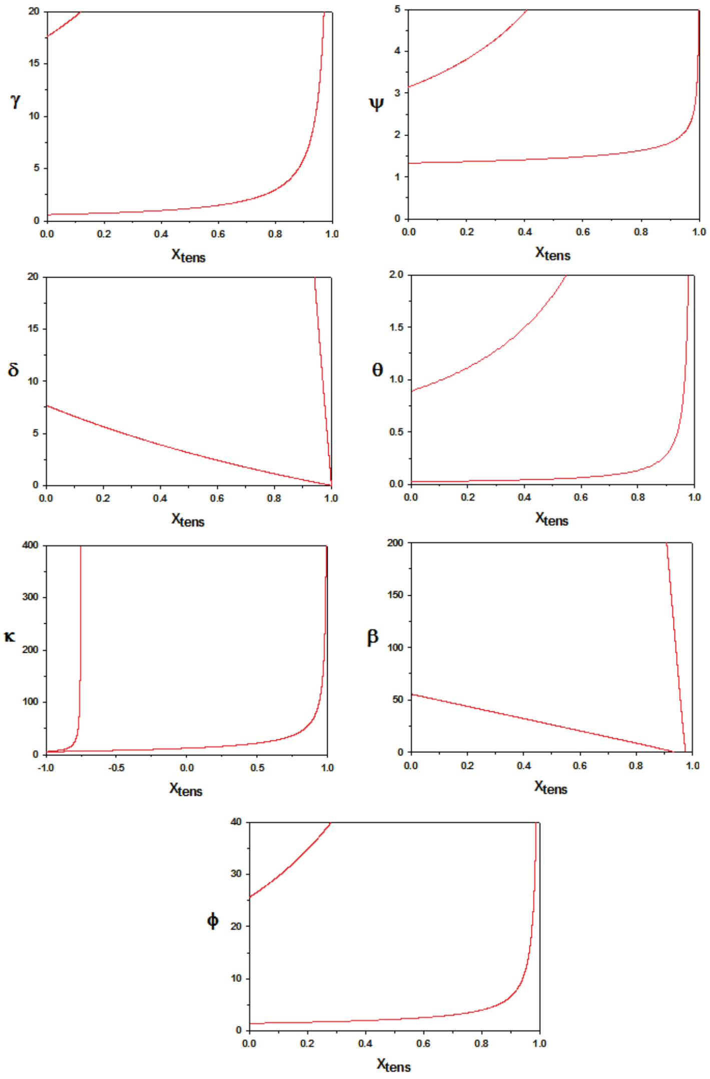

Supplementary Figure S3. Two-parameter bifurcation diagrams for the CycB-MCC-APC network illustrated in Fig. 5D and defined by Equations (S3.2)-(S3.6). For the dimensionless parameter indicated on the vertical axis in each panel, the checkpoint control mechanism is bistable in the region between the two red curves. For the mitotic checkpoint to be both sensitive and irreversible, the control mechanism must be bistable in the region $0 < X_{\mathrm{tens}} < 0.95$. This requirement puts constraints on the acceptable ranges of the seven dimensionless parameters, as summarized in Table S1.

Supplement 4. Dynamical Systems Theory

Phase Plane Diagrams

A set of nonlinear ODEs for a reaction network on N biochemical species defines a vector field in the network's state space spanned by the concentration variables $X_1, X_2, \ldots, X_N$. If $N = 2$, then the rate equations have the form

$$\frac{d}{dt}X_1 = F_1(X_1, X_2), \; \frac{d}{dt}X_2 = F_2(X_1, X_2) \qquad \text{(S4.1)}$$

and the state space is a plane. We can visualize the vector field by drawing a "phase plane portrait", as in Fig. 3C and 3D. On the phase plane, spanned by X_1 and X_2, we plot two curves (called "balance curves" or "nullclines") defined by the algebraic equations:

$$F_1(X_1, X_2) = 0, \; F_2(X_1, X_2) = 0 \qquad \text{(S4.2)}$$

On the first curve, $dX_1/dt = 0$ and the vector field is vertical (i.e., there is no change in the horizontal—X_1—direction). On the second curve, $dX_2/dt = 0$ and the vector field is horizontal (i.e., there is no change in the vertical—X_2—direction). The intersections of these curves correspond to steady state solutions of the dynamical system (i.e., points where neither X_1 nor X_2 are changing with time). As illustrated in Figs. 3C and 3D, phase plane portraits can be quite informative in locating steady states and determining their stability. In these figures, the filled circles represent stable steady states (small perturbations away from the steady state return to the vicinity of the filled circle), whereas the open circle represents an unstable steady state (small perturbations away from the steady state are amplified, and—in this case—trajectories move off in search of a stable steady state).

One-parameter Bifurcation Diagrams

The existence of stable and unstable steady-state solutions of a nonlinear dynamical system, as defined in the previous subsection, depend on the precise values of the rate constants (called "parameters") that appear on the right-hand-sides of the rate equations. We make these parameters explicit by rewriting Equations (S4.1) and (S4.2) as

$$\frac{d}{dt}X_1 = F_1(X_1, X_2; p_1, p_2), \; \frac{d}{dt}X_2 = F_2(X_1, X_2; p_1, p_2) \qquad \text{(S4.1}')$$

$$F_1(X_1, X_2; p_1, p_2) = 0, \; F_2(X_1, X_2; p_1, p_2) = 0 \qquad \text{(S4.2}')$$

Typically, a biochemical reaction network will be described by many variables and many parameters, but we need only keep track of two for now. We would like to know how the steady-state solutions, the values of X_1 and X_2 that satisfy Equations (S4.2′) simultaneously, move around as the parameters, p_1 and p_2, are varied.

First, we fix p_2 and vary only p_1. (This is called a "one-parameter" bifurcation diagram because we vary only one parameter; all other parameters are fixed.) For each value of p_1 we determine the steady state values of X_1 and X_2 and we choose to plot one of them (it doesn't matter which one) as a function of p_1. For example, in Fig. 2B we plot the steady state value of free activator, A_{ss}, as a function of varying levels of total inhibitor concentration, I_T, which is a parameter in Equations (1)-(6). The one-parameter bifurcation diagram can be thought of as a "signal-response curve". The total amount of inhibitor is the signal, and the steady-state activity of A is the control system's response. In this case (Fig. 2B) there is a range of signal levels, $2.4 < I_T < 5.7$, for which the response is bistable (two possible, stable, steady state levels of active A). The endpoints of this range are called "bifurcation points", because the qualitative behavior of the dynamical system changes abruptly as the parameter value crosses the endpoints. For example, as I_T decreases from just above 2.4 to just below 2.4, the system loses a stable steady state (the lower stable steady state coalesces with the intermediate unstable steady state), and the system now has only one stable steady state (the upper one). If the control system started in the lower stable steady state (as in path *b* in Fig. 2B), then as I_T drops below 2.4 the control system must make an abrupt transition to the upper steady state. The two bifurcation points in this figure, at $I_T = 2.4$ and 5.7, are called "saddle-node" bifurcation points because at these points the control system gains or loses a pair of steady states: a stable steady state (called a "node") and an unstable steady state (called a "saddle point").

Two-parameter Bifurcation Diagrams

The function of a two-parameter bifurcation diagram is to track how these saddle-node bifurcation points change as a second parameter, p_2, of the control system is varied. An example is provided by Fig. 2D: the primary bifurcation parameter is I_T, and the secondary bifurcation parameter is A_T. For $A_T = 1$ we can see (on Fig. 2D) the locations of the two saddle-node bifurcations at $I_T = 2.4$ and 5.7. We can also see how, as we vary A_T away from 1, the locations of the two bifurcation points change. The region of bistability expands as we increase A_T and

shrinks as we decrease A_T. The two boundaries of the bistable region come together at a cusp point at $I_T = 0.4$, $A_T = 0.2$.

We refer to two-parameter bifurcation diagrams as “control planes”. The two parameters (I_T and A_T in the case of Fig. 2D) provide controlling inputs into the reaction network. The control plane diagram summarizes the qualitative response (monostable or bistable) of the network to the controlling signals.

- - -

Supplement 5. ODE Files

```
#Fig 2B, Fig.2C & Fig.2D
A'  = - kas1*A*I + kdi1*C + kcat1*C - kas2*A*IM + kdi2*CM + kcat2*CM
IM' = - kas2*A*IM + kdi2*CM + kcat1*C - kdm1*IM + kdm2*IMM
IMM'= kcat2*CM - kdm2*IMM
C'  = kas1*A*I - kdi1*C - kcat1*C
CM  = AT-A-C
I   = IT-C-IM-CM-IMM
par IT=0, AT=1, kas1=100, kas2=50, kdi1=0.5, kdi2=1
par kcat1=0.5, kcat2=50, kdm1=1, kdm2=1
# use parameter values of AT=0 and IT=4 for Fig.2C and change their order in the parameter list
@ total=500,dt=0.25,meth=STIFF,xlo=0,xhi=500,ylo=0,yhi=10
@ NTST=15,NMAX=100000,NPR=100000,DS=0.01
@ DSMAX=0.01,DSMIN=0.0001,PARMIN=0,PARMAX=10
@ AUTOXMIN=0,AUTOXMAX=7,AUTOYMIN=0,AUTOYMAX=1
# use AUTOXMAX=2, AUTOYMAX=2 for Fig.2C
# use AUTOXMAX=10, AUTOYMAX=4 for Fig.2D
done
```

```
#Fig 3B
A'  = - kas1*A*I + kdi1*C + kcat1*C
IM' = - km*A*IM - kdm1*IM + kdm2*IMM + kcat1*C
IMM'= km*A*IM - kdm2*IMM
C   = AT-A
I   = IT-C-IM-IMM
par IT=0.01, AT=1, kas1=100, kdi1=0.5, km=50
par kcat1=0.5, kdm1=1, kdm2=1
@ total=500,dt=0.25,meth=STIFF,xlo=0,xhi=500,ylo=0,yhi=2
@ NTST=12,NMAX=100000,NPR=100000,DS=0.01
@ DSMAX=0.01,DSMIN=0.0001,PARMIN=0,PARMAX=10
@ AUTOXMIN=0,AUTOXMAX=10,AUTOYMIN=0,AUTOYMAX=4
done
```

```
#Fig 3C & Fig.3D
AI = 2*AT*IH/(AT+IH+Km1 +sqrt((AT+IH+Km1)^2-4*AT*IH))
A = AT - AI
IM = IT - IH - IMM
dIH/dt  = kdm1*IM - kcat1*AI
dIMM/dt = kas2*A*IM - kdm2*IMM
par AT=2, IT=6, Km1=0.01
par kcat1=0.5, kas2=50, kdm1=1, kdm2=1
# use AT=3 for Fig.3D
@ xp=IH, yp=IMM
@ total=400,dt=0.5,meth=STIFF
@ xlo=0,xhi=6,ylo=0,yhi=6,NMESH=400
done
```

```
#Fig 4B & Fig.4D
# Fix ClnT=0 for Fig 4B
# Use ClnT as second bifurcation parameter for Fig 4D and make DS negative
init SICT=4,ClbSic=0,ClnSic=0,SICP=0
SICT' = ks - kd*SICT - kp_b*SICP*Clb - kp_n*SICP*Cln
ClbSic' = kas_b*SIC*Clb - (kdi_b + kd + kcat_b)*ClbSic
ClnSic' = kas_n*SIC*Cln - (kdi_n + kd + kcat_n)*ClnSic
SICP' = kcat_b*ClbSic + kcat_n*ClnSic- (kdp+kd)*SICP-kp_b*SICP*Clb-kp_n*SICP*Cln
SIC = SICT - ClbSic - ClnSic - SICP
Clb = ClbT - ClbSic
Cln = ClnT - ClnSic
par ClbT=0, ClnT=0, kcat_b=0.5, kcat_n=2
par kas_b=100, kas_n=2, kdi_b=0.01, kdi_n=2, kp_b=20, kp_n=0.1
par ks=0.2, kd=0.05, kdp=0.5
@ total=500,dt=.25,meth=STIFF,xlo=0,xhi=500,ylo=0,yhi=10
@ NTST=10,NMAX=10000,NPR=10000,DS=0.01
@ DSMAX=.01,DSMIN=.001,PARMIN=-1,PARMAX=10
@ AUTOXMIN=0,AUTOXMAX=1,AUTOYMIN=0,AUTOYMAX=4
done
```

```
#Fig 4E
init SICT=4,ClbSic=0,ClnSic=0,SICP=0
ClnT'  = kscln*(1-heav(t-Noff))-kdcln*heav(t-Noff)*ClnT
ClbT'  = ksclb*(1-heav(t-Boff))-kdclb*heav(t-Boff)*ClbT - kdclb2*ClbT
SICT'  = ks - kd*SICT - kp_b*SICP*Clb - kp_n*SICP*Cln
ClbSic'= kas_b*SIC*Clb - (kdi_b + kd + kcat_b)*ClbSic
ClnSic'= kas_n*SIC*Cln - (kdi_n + kd + kcat_n)*ClnSic
SICP'= kcat_b*ClbSic+kcat_n*ClnSic-(kdp + kd)*SICP-kp_b*SICP*Clb-kp_n*SICP*Cln
SIC    = SICT - ClbSic - ClnSic - SICP
Clb    = ClbT - ClbSic
Cln    = ClnT - ClnSic
aux Clb    = ClbT - ClbSic
par kscln=0.065, ksclb=0.065, kdcln=0.2, kdclb=0.5
par kdclb2=0.02, kcat_b=0.5, kcat_n=2
par kas_b=100, kas_n=2, kdi_b=0.01, kdi_n=2, kp_b=20, kp_n=0.1
par ks=0.2, kd=0.05, kdp=0.5, Noff=30, Boff=50
@ total=100,dt=0.25,bound=10000,meth=stiff
@ xlo=0,xhi=70,ylo=0,yhi=4
@ NPLOT=4, yp1=SICT, yp2=ClnT, yp3=ClbT, yp4=Clb
done
```

```
# Fig.5B
init MCCT=1.98, MCCU=0.09
MCCT' = ka*Nt*(1-Xtens)*(Mad2T - MCCT) - ku*APC*MCCU
MCCU'= kcat*APCMCC - (kdu +ku*APC)*MCCu
BB = APCT + MCCT - MCCU + Km
APCMCC = 2*APCT*(MCCT-MCCU)/(BB+sqrt(BB^2-4*APCT*(MCCT-MCCU)))
APC = APCT-APCMCC
par Xtens=0, APCT=1, Nt=1, Mad2T=2
par ka=1, kcat=0.5, ku=10, kdu=5, Km=0.01
@ total=400,dt=0.5,meth=STIFF
@ xlo=0,xhi=400,ylo=1,yhi=3
@ NTST=15,NMAX=200000,NPR=10000,DS=0.02
@ DSMAX=0.01,DSMIN=0.001,PARMIN=-10,PARMAX=1
@ AUTOXMIN=0.8,AUTOXMAX=1,AUTOYMIN=0,AUTOYMAX=2
done
```

```
# Fig.5C
init MCCT=1.99, MCCU=0.09, Sec=1.8, Xtens=1
MCCT' = ka*Nt*(1-Xtens)*(Mad2T - MCCT) - ku*APC*MCCU
MCCU' = kcat*APCMCC - (kdu + ku*APC)*MCCU
Sec' = ksec - (kdsec + kdsec_apc*APC)*Sec
Xtens'=-ktens*heav(t-Noff)*Xtens
BB = APCT + MCCT - MCCU + Km
APCMCC = 2*APCT*(MCCT-MCCU)/(BB+sqrt(BB^2-4*APCT*(MCCT-MCCU)))
APC = APCT - APCMCC
aux APCMCC = APCMCC
aux APC = APC
par Nt=1, ka=1, APCT=1, Mad2T=2
par kcat=0.5, ku=10, kdu=5, Km=0.01
par ksec=0.1, kdsec=0.05, kdsec_apc=0.5
par ktens=0.02, Noff=60
@ total=100,dt=0.5,meth=STIFF
@ xlo=0,xhi=100,ylo=0,yhi=2
@ NPLOT=5, yp1=MCCT, yp2=APCMCC, yp3=Sec, yp4=Xtens, yp5=APC
done
```

```
# Fig.5E
# MCCT= MCC + APCMCC + MCCU
init MCCT=1.99, APCMCC=0.99, MCCU=0.09, CycB=0.47, X0A=0.19
MCCT' = ka*Nt*X0A*(Mad2T - MCCT) - ku*APC*MCCU
APCMCC' = kas*(APCT - APCMCC)*(MCCT - APCMCC-MCCU) - (kdi + kcat)*APCMCC
MCCU' = kcat*APCMCC - (kdu + ku*APC)*MCCU
CycB' = kscyc - (kdcyc' + kdcyc*APC)*CycB
X0A' = kan*CycB*(1-Xtens-X0A)-kin*CAPP*X0A
APC = APCT - APCMCC
aux APC = APC
par Xtens=0, Nt=1, ka=5, APCT=1, Mad2T=2
par kcat=0.5, ku=10, kdu=5
par kas=100, kdi=0.5
par kscyc=0.01, kdcyc'=0.01, kdcyc=1
par kan=1,kin=2, CAPP=1
@ total=400,dt=0.5,meth=STIFF
@ xlo=0,xhi=400,ylo=1,yhi=3
@ NTST=15,NMAX=2000,NPR=100,DS=0.02
@ DSMAX=0.01,DSMIN=0.001,PARMIN=-10,PARMAX=1
@ AUTOXMIN=0,AUTOXMAX=1,AUTOYMIN=0,AUTOYMAX=2
done
```

```
# Fig.5F
# MCCT= MCC + APCMCC + MCCU
init MCCT=1.99, APCMCC=0.98, MCCU=0.09, CycB=0.47, X0A=0.19, Xtens=1
MCCT' = ka*Nt*X0A*(Mad2T - MCCT) - ku*APC*MCCU
APCMCC' = kas*(APCT - APCMCC)*(MCCT - APCMCC-MCCU) - (kdi + kcat)*APCMCC
MCCU' = kcat*APCMCC - (kdu + ku*APC)*MCCU
CycB' = kscyc - (kdcyc' + kdcyc*APC)*CycB
X0A' = kan*CycB*(1-Xtens-X0A)-kin*CAPP*X0A
Xtens'=-ktens*heav(t-Noff)*Xtens
APC = APCT - APCMCC
aux APC = APC
par Nt=1,ka=5, APCT=1, Mad2T=2
par kcat=0.5, ku=10, kdu=5
par kas=100, kdi=0.5
par kscyc=0.01, kdcyc'=0.01, kdcyc=1
par kan=1,kin=2,CAPP=1
par ktens=0.02, Noff=60
@ total=100,dt=0.5,meth=STIFF
@ xlo=0,xhi=100,ylo=0,yhi=2
@ NPLOT=5, yp1=MCCT, yp2=APCMCC, yp3=CycB, yp4=Xtens, yp5=APC
done
```

Supplementary Table

Table S1. Parameter ranges that ensure irreversibility of the mitotic checkpoint

	Present Model		Model of He et al. (2011)	
Parameter	Definition	Range	Definition	Range
Θ = 0.5	$\frac{k_{\text{an}} k_{\text{scyc}} / k_{\text{dcyc}}}{k_{\text{in}} [\text{CAPP}]}$	0.5—0.9	same	1—9.5
Ψ = 2	$\frac{[\text{Mad2T}]}{[\text{APCT}]}$	2—3.1	same	2—14
β = 100	$\frac{k_{\text{dcyc\_apc}} [\text{APCT}]}{k_{\text{dcyc}}}$	55—100	same	15—500
γ = 10	$\frac{k_{\text{a}} N_{\text{T}}}{k_{\text{cat}}}$	10—17.5	comparable	50—800
κ = 100	$\frac{k_{\text{as}} [\text{APCT}]}{k_{\text{di}} + k_{\text{cat}}}$	> 100	comparable	> 100
δ = 20	$\frac{k_{\text{u}} [\text{APCT}]}{k_{\text{cat}}}$	7.5—20	$\frac{k_{\text{amad,apc}} [\text{APCT}]}{k_{\text{imad}}}$	10—400
φ = 10	$\frac{k_{\text{du}}}{k_{\text{cat}}}$	10—26	not relevant	
